# Evidence for Genetic Correlations and Bidirectional, Causal Effects Between Smoking and Sleep Behaviors

**DOI:** 10.1093/ntr/nty230

**Published:** 2018-10-26

**Authors:** Mark Gibson, Marcus R Munafò, Amy E Taylor, Jorien L Treur

**Affiliations:** 1 School of Experimental Psychology, University of Bristol, Bristol, UK; 2 MRC Integrative Epidemiology Unit, University of Bristol, Bristol, UK; 3 UK Centre for Tobacco and Alcohol Studies, Bristol, UK; 4 Population Health Sciences, Bristol Medical School, University of Bristol, Bristol, UK; 5 NIHR Biomedical Research Centre, University Hospitals Bristol NHS Foundation Trust and University of Bristol, UK

## Abstract

**Introduction:**

Cigarette smokers are at increased risk of poor sleep behaviors. However, it is largely unknown whether these associations are due to shared (genetic) risk factors and/or causal effects (which may be bidirectional).

**Methods:**

We obtained summary-level data of genome-wide association studies of smoking (smoking initiation [*n* = 74 035], cigarettes per day [*n* = 38 181], and smoking cessation [*n* = 41 278]) and sleep behaviors (sleep duration and chronotype, or “morningness” [*n* = 128 266] and insomnia [*n* = 113 006]). Using linkage disequilibrium (LD) score regression, we calculated genetic correlations between smoking and sleep behaviors. To investigate causal effects, we employed Mendelian randomization (MR), both with summary-level data and individual-level data (*n* = 333 581 UK Biobank participants). For MR with summary-level data, individual genetic variants were combined with inverse variance–weighted meta-analysis, weighted median regression, MR-Robust Adjusted Profile Score, and MR Egger methods.

**Results:**

We found negative genetic correlations between smoking initiation and sleep duration (*r*g = −.14, 95% CI = −0.26 to −0.01) and smoking cessation and chronotype (*r*g = −.18, 95% CI = −0.31 to −0.06), and positive genetic correlations between smoking initiation and insomnia (*r*g = .27, 95% CI = 0.06 to 0.49) and cigarettes per day and insomnia (*r*g = .15, 95% CI = 0.01 to 0.28). MR provided strong evidence that smoking more cigarettes causally decreases the odds of being a morning person, (RAPS) and weak evidence that insomnia causally increases smoking heaviness and decreases smoking cessation odds.

**Conclusions:**

Smoking and sleep behaviors show moderate genetic correlation. Heavier smoking seems to causally affect circadian rhythm and there is some indication that insomnia increases smoking heaviness and hampers cessation. Our findings point to sleep as a potentially interesting smoking treatment target.

**Implications:**

Using LD score regression, we found evidence that smoking and different sleep behaviors (sleep duration, chronotype (morningness), and insomnia) are moderately genetically correlated—genetic variants associated with less or poorer sleep also increased the odds of smoking (more heavily). MR analyses suggested that heavier smoking causally affects circadian rhythm (decreasing the odds of being a morning person) and there was some indication that insomnia increases smoking heaviness and hampers smoking cessation. Our findings indicate a complex, bidirectional relationship between smoking and sleep behaviors and point to sleep as a potentially interesting smoking treatment target.

## Introduction

Observationally, cigarette smoking is associated with poor sleep. Smokers take a longer time to fall asleep and are at higher risk of experiencing sleep disturbances.^1^ Although sleep duration is generally shorter in smokers, longer than average sleep duration is more common too.^2,3^ One longitudinal study that followed substance naïve adolescents into early adulthood found that erratic sleep patterns predicted smoking initiation.^4^ In adults, transitioning from “adequate” to “inadequate” sleep duration over a period of 5 years predicted heavier smoking^5^ and preexisting insomnia symptoms increased the likelihood of relapse after an attempt to quit smoking.^6^ Chronotype—being a “morning” versus an “evening” person—has also been linked to smoking such that smokers are more likely to be an evening person.^7^ This is in contrast to evidence suggesting that young adolescents with an evening chronotype show a lower odds of smoking initiation 4–5 years later.^4^ The observational nature of the studies described here precludes strong conclusions about causality. Unraveling the nature of the relationship between smoking and poor sleep is important, given the major health burden that both behaviors pose.^8,9^

Observational associations between smoking and sleep may reflect common risk factors. These could be environmental in nature, such as socioeconomic factors,^10,11^ or genetic—twin and family studies have reported a moderate-to-high heritability for both smoking and sleep.^2,12,13^ Genome-wide association studies (GWAS) have identified genetic variants robustly associated with smoking initiation, number of cigarettes smoked per day, and smoking cessation,^14^ and more recently, sleep duration and chronotype^15^ and insomnia.^16^ This knowledge of the genetic architecture of smoking and sleep allows us to investigate the degree to which genetic risk for both phenotypes overlaps. With linkage disequilibrium (LD) score regression, a genetic correlation between two phenotypes can be calculated such that “0” reflects no overlap in genetic risk and “1” reflects that genetic risk for both phenotypes is exactly the same.^17^ Hammerschlag et al.^16^ reported sizeable, positive, genetic correlations between smoking and insomnia complaints, but whether smoking behavior shows genetic overlap with sleep duration and chronotype is still unknown. Moreover, genetic correlations may also reflect causal relationships. If, for instance, smoking cigarettes causally increases insomnia, then genetic variants that underlie vulnerability for smoking will also be associated with insomnia.

Causal effects could operate in either direction, from smoking to poor sleep (possibly due to nicotine’s stimulating effects) and from poor sleep to smoking (such that cigarettes are used as self-medication against fatigue). A meta-analysis of clinical trials that looked at the effectiveness of nicotine patches as an aid to quit smoking found that patch users experienced more sleep disturbances than controls. The effects were proportional to the strength of the patch, and worse when it was left on overnight,^18^ suggesting that nicotine has a causal, negative effect on sleep. This is in line with research showing that nicotine can inhibit sleep-promoting neurons, thereby causing arousal and changes in electroencephalography sleep waves.^19,20^ In the other direction, smokers who were offered cigarettes or money picked cigarettes more often when they were sleep deprived, even when the monetary value was greater than that of the cigarette.^21^ Finally, animal work has suggested that passive smoking, via an effect on gene expression, can alter circadian rhythm (which determines chronotype).^22^ Overall, current findings are mixed and have focused on short-term rather than long-term effects. Novel methods are needed to fully disentangle the complex relationship between smoking and sleep. To distinguish genetic correlation from causal relationships, Mendelian randomization (MR) analysis can be applied. MR infers causality by taking a set of genetic variants robustly associated with an exposure variable as a proxy for this exposure and estimating its causal effect on an outcome variable.^23^ Potential horizontal pleiotropy (genetic variants affecting the outcome directly, not acting through the exposure) can be assessed with sensitivity analyses.

In this study, we first calculated genetic correlations between smoking (smoking initiation, cigarettes smoked per day, and smoking cessation), and sleep behaviors (sleep duration, chronotype, and insomnia) based on summery-level data of large, published GWAS. We then employed MR—using individual-level data of 333 581 participants of UK Biobank and summary-level data—to test bidirectional, causal effects between smoking and sleep behaviors.

## Methods

### Data Sources

For LD-score regression and MR with summary-level data (two-sample MR), we used summary statistics from GWAS on smoking initiation (*n* = 74 035), cigarettes smoked per day (*n* = 38 181), and smoking cessation (being a former vs. a current smoker; *n* = 41 278) from the Tobacco and Genetics consortium.^14^ GWAS based on the first release of UK Biobank were used for sleep duration (measured in hours and as undersleeping [≤6 hours vs. 7–8 hours] and oversleeping [≥9 hours vs. 7–8 hours]), chronotype (measured on a five-point scale with 2 coded as being a “morning person” and −2 being an “evening person”; *n* = 128 266),^15^ and insomnia (usually having trouble falling asleep at night or waking up in the middle of the night [cases] vs. never or rarely or sometimes having these problems [controls]; *n* = 113 006).^16^ Where there was evidence for causal effects of any of the sleep behaviors on smoking, we sought replication using the second release of UK Biobank as the outcome sample (*n* = 241 008 for smoking initiation; *n* = 67 193 for cigarettes per day; *n* = 107 874 for smoking cessation).

For MR with individual-level data (one-sample MR), we obtained data of 333 581 participants of UK Biobank (first + second release).^24^ Details on the UK Biobank sample and quality control of the genetic data within the Medical Research Council - Integrative Epidemiology Unit are available in the Supplementary Material.

### Statistical Analysis

#### LD-Score Regression

Genetic correlations between smoking and sleep behaviors were estimated with LD-score regression.^17^ This method is based on the expected relationship between the degree of LD between single nucleotide polymorphisms (SNPs) and the strength of their association with the phenotype in question as derived from GWAS. The GWAS estimate for a particular SNP incorporates the effects of all other SNPs that are in LD with that SNP. Logically, SNPs that are in high LD with many neighboring SNPs have a higher chance of tagging a causal genetic variant as compared to SNPs in lower LD. From this it follows that SNPs with a higher degree of LD with neighboring SNPs show larger test statistics. This information is used to compute a genetic correlation between two phenotypes. For a more detailed description of LD-score regression, we refer to the work of Bulik-Sullivan et al.^17^

#### MR With Individual-Level Data

We investigated causal effects of *cigarettes smoked per day* and *smoking cessation* on sleep behaviors using MR with individual-level data. Because genetic variants previously associated with cigarettes smoked per day and smoking cessation were identified in samples of smokers,^14^ MR analyses investigating causal effects of these variables as exposures have to be stratified by smoking status. For cigarettes smoked per day, we used the genetic variant rs16969968 as instrumental variable—the minor allele of this SNP is associated with smoking, on average, one additional cigarette per day.^25^ Analyses were performed in never, former, and current smokers separately. For smoking cessation, we used the genetic variant rs3025343 as instrumental variable. The major allele of this variant has been associated with a higher odds of being a former versus a current smoker.^14^ Analyses were performed in ever-smokers (current + former smokers) and never-smokers separately. Associations between the genetic instruments and the exposure variables as well as common confounding variables are provided in the Supplementary Material (Supplementary Tables 1–7).

MR entailed linear and logistic regression analyses performed in STATA. The genetic instrument for the smoking (exposure) variable was the independent variable (coded as 0, 1, or 2 risk alleles) whereas the sleep (outcome) variable was the dependent variable. If, for instance, the genetic instrument for smoking more cigarettes per day predicts sleep problems in smokers, but not in nonsmokers, this would suggest a causal effect of smoking on sleep.

#### Two Sample MR With Summary-Level Data

Next, we investigated causal effects for all other relationships between smoking and sleep, using MR with summary-level data.^26^ In this approach, the gene-exposure association and the gene-outcome association are obtained from two different samples. Genetic instruments for the exposure variables were selected in the exposure GWAS at two levels of significance, first using SNPs reaching genome-wide significance (*p* < 5 × 10^−8^) and second using a more liberal threshold of *p* less than 1 × 10^−5^ (independent SNPs were identified by pruning on *r*^2^ < .001). Gene-outcome associations, for SNPs associated with the exposure, were then extracted from the outcome GWAS. When SNPs were not available in the outcome GWAS, proxies were used (LD *R*^2^ > 0.8; identified using online tool SNiPa). A full list of the SNPs used in each analysis is provided in Supplementary Table 8.

Analyses were conducted using the R package of MR-Base, a database and analytical platform to perform MR^27^. Causal estimates were calculated using the Wald ratio in case of genetic instruments consisting of a single SNP.^26^ Where multiple SNPs were available, these were combined using inverse variance−weighted fixed effects meta-analysis (IVW).^28^ The IVW estimate is the mean average of the Wald ratios of all SNPs, inversely weighted by their standard error. Conveniently, two-sample MR allows sensitivity analyses that are more robust to horizontal pleiotropy, albeit less powerful. We employed three sensitivity analyses, relying on distinct and contrasting assumptions; weighted median regression, MR-RAPS (Robust Adjusted Profile Score) and MR-Egger regression. Weighted median regression is able to provide a consistent estimate of a causal effect even when up to 50% of the weight in a polygenic score comes from invalid instruments.^29^ MR-RAPS is an extension of IVW, which is more robust to deviations of the underlying assumptions of MR because it adjusts the profile likelihood of the summary data that is used.^30^ MR-Egger regression is a variation of a test used in meta-analyses to assess small study bias. It rests on the InSIDE (Instrument Strength Independent of Direct Effect) assumption, which means that the strength of the instrument should not correlate with the direct effect that the instrument has on the outcome—a much weaker assumption than that of no horizontal pleiotropy.^31^ The intercept of MR-Egger indicates whether there is horizontal pleiotropy. To quantify heterogeneity between genetic variants and indicate how likely it is that the NOME (NO Measurement Error) assumption was violated, the *I*^2^ statistic was computed. If *I*^2^ is less than 0.9 the NOME assumption is likely to be violated. In that case, we applied a SIMEX (simulation extrapolation) correction to MR-Egger, which adjusts for bias caused by such violation.^32^ MR-RAPS and MR-Egger were only reported for genetic instruments that contained a sufficient number of SNPs (≥10).

## Results

**Figure 1.**
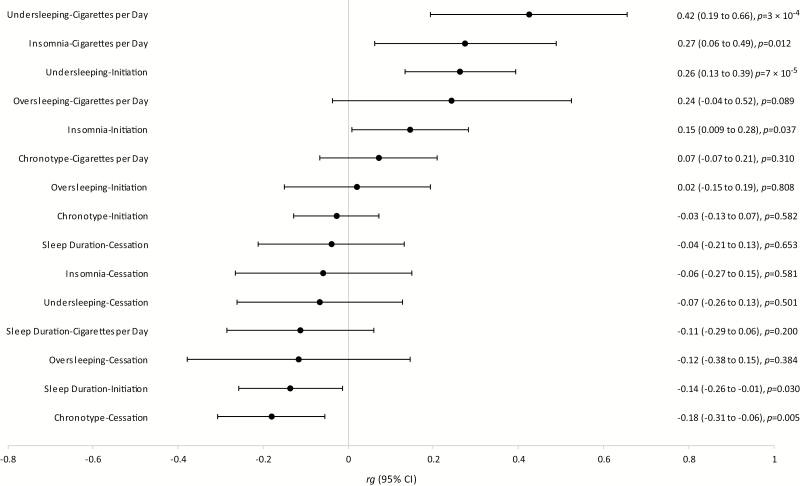
Forest plot depicting genetic correlations (*r*g), as calculated with linkage disequilibrium (LD) score regression, between smoking behaviors and sleep behaviors.

The results of LD-score regression are presented as a forest plot in Figure 1. We observed a negative correlation between sleep duration and smoking initiation (*r*g = −.14, 95% CI = −0.26 to −0.01, *p* = .030) and, in agreement with this, a positive genetic correlation between undersleeping and smoking initiation (*r*g = .26, 95% CI = 0.13 to 0.39, *p* = 7 × 10^−5^). The strongest correlation we observed was between undersleeping and cigarettes per day, (*r*g = .42, 95% CI = 0.19 to 0.66, *p* = 3 × 10^−4^), which, together with the other correlations described here, points to less or poorer sleep being associated with smoking (more heavily). Finally, we observed a negative correlation between chronotype and smoking cessation (*r*g = −.18, 95% CI = −0.31 to −0.06, *p* = .005), indicating that genetic variants associated with being a morning person are also associated with reduced likelihood of quitting smoking. As reported previously,^16^ we found positive genetic correlations between insomnia and cigarettes per day (*r*g = .27, 95% CI = 0.06 to 0.49, *p* = .012) and insomnia and smoking initiation (*r*g = .15, 95% CI = 0.009 to 0.28, *p* = .037). There was no clear evidence for genetic correlations between the other sleep and smoking behaviors.

### MR With Individual-Level Data

The minor allele of rs16969968, which is associated with smoking more cigarettes per day, was strongly associated with a lower chronotype score (ie, being more of an “evening” person) in current smokers (beta = −.062, 95% CI = −0.084 to −0.039, *p* = 6.9 × 10^−8^; Table 1). A weaker effect size, in the same direction, was found in former smokers (beta = −.016, 95% CI = −0.027 to −0.005, *p* = .004). In never-smokers, the effect was in the opposite direction, with the smoking increasing allele of rs16969968 being associated with being a “morning” person (beta = .011, CI = 0.003 to 0.020, *p* = .01). There was no clear evidence for causal effects of smoking heaviness on sleep duration (in hours of sleep or measured as undersleeping or oversleeping) or insomnia, nor was there clear evidence for causal effects of smoking cessation on sleep behaviors.

**Table 1.** Mendelian Randomization (MR) Analyses With Individual-Level Data, Estimating Causal Effects of Smoking Behaviors (Cigarettes per Day and Smoking Cessation) on Sleep Behaviors (Sleep Duration, Chronotype, and Insomnia)

Exposure	Outcome	Smoking status	*N*	Beta/OR (95% CI)	*p* value
rs16969968 (instrument for cigarettes per day)	Sleep duration	Never	183 333	−0.006 (−0.013 to 0.002)	.13
		Former	117 743	0.009 (−0.001 to 0.018)	.07
		Current	33 101	−0.014 (−0.034 to 0.006)	.17
	Undersleeping	Never	170 271	1.01 (0.99 to 1.03)	.12
		Former	108 096	0.99 (0.97 to 1.01)	.24
		Current	30 237	1.02 (0.98 to 1.05)	.37
	Oversleeping	Never	141 833	0.99 (0.96 to 1.01)	.30
		Former	89 716	1.01 (0.98 to 1.05)	.41
		Current	23 486	0.98 (0.93 to 1.04)	.58
	Chronotype	Never	183 925	0.011 (0.003 to 0.020)	.01
		Former	118 041	−0.016 (−0.027 to −0.005)	.004
		Current	33 285	−0.062 (−0.084 to −0.039)	6.9 × 10^−8^
	Insomnia	Never	184 184	0.99 (0.98 to 1.01)	.38
		Former	118 181	1.01 (0.99 to 1.02)	.56
		Current	33 343	1.00 (0.97 to 1.04)	.79
rs3025343 (instrument for smoking cessation)	Sleep duration	Never	183 333	−0.007 (−0.018 to 0.003)	.18
		Ever	117 743	0.005 (−0.009 to 0.018)	.52
	Undersleeping	Never	170 271	1.01 (0.99 to 1.04)	.34
		Ever	108 096	1.01 (0.98 to 1.04)	.47
	Oversleeping	Never	141 833	0.99 (0.95 to 1.03)	.54
		Ever	89 716	1.04 (0.99 to 1.09)	.11
	Chronotype	Never	183 925	−0.002 (−0.015 to 0.010)	.72
		Ever	118 041	0.011 (−0.006 to 0.027)	.20
	Insomnia	Never	183 082	1.01 (0.99 to 1.03)	.37
		Ever	118 181	1.00 (0.98 to 1.03)	.85

For cigarettes per day, coefficients represent the change in outcome per additional minor allele of rs16969968. For smoking cessation, coefficients represent the change in outcome per additional major allele of rs3025343. Sleep duration was measured in hours per day. Undersleeping was defined as sleeping ≤6 hours versus 7–8 hours and oversleeping as ≥9 hours versus 7–8 hours. Chronotype was measured on a five-point scale, with higher scores indicating being more of a “morning person” and lower scores more of an “evening person.” Insomnia was measured as usually having trouble falling asleep at night or waking up in the middle of the night (cases) versus never or rarely or sometimes having these problems (controls).

### MR With Summary-Level Data

Using MR with summary-level data of published GWAS studies on smoking and sleep behaviors, there was no clear evidence for a causal influence of smoking initiation on sleep duration, chronotype, or insomnia (Table 2).

**Table 2.** Two-Sample Mendelian Randomization (MR) Analyses With Summary-Level Data, Estimating Causal Effects of Smoking Initiation on Sleep Behaviors (Sleep Duration, Chronotype, and Insomnia)

Exposure	Outcome	Threshold	*N* SNPs	IVW		Weighted median		MR-RAPS		MR-Egger	
				Beta/OR (95% CI)	*p*	Beta/OR (95% CI)	*p*	Beta/OR (95% CI)	*p*	Beta/OR (95% CI)	*p*
Smoking initiation	Sleep duration	*p* < 1 × 10^−5^	20	−0.01 (−0.04 to 0.02)	.61	−0.004 (−0.05 to 0.04)	.86	−0.01 (−0.05 to 0.03)	.63	0.02 (−0.08 to 0.12)	.77
Smoking initiation	Undersleeping	*p* < 1 × 10^−5^	19	1.01 (0.99 to 1.02)	.13	1.01 (0.99 to 1.03)	.50	1.01 (1.00 to 1.03)	.14	1.00 (0.97 to 1.03)	.99
Smoking initiation	Oversleeping	*p* < 1 × 10^−5^	19	1.00 (0.99 to 1.01)	.92	1.01 (0.96 to 1.03)	.36	1.00 (0.99 to 1.01)	.93	1.01 (0.96 to 1.06)	.63
Smoking initiation	Chronotype	*p* < 1 × 10^−5^	20	0.001 (−0.04 to 0.04)	.97	−0.02 (−0.06 to 0.03)	.49	−0.003 (−0.04 to 0.04)	.90	−0.09 (−0.22 to 0.04)	.19
Smoking initiation	Insomnia	*p* < 1 × 10^−5^	20	1.00 (0.92 to 1.08)	.99	1.01 (0.91 to 1.12)	.82	1.00 (0.93 to 1.08)	.99	1.20 (0.96 to 1.51)	.13

Coefficients represent the change in outcome per 2.72-fold increase in the prevalence of smoking initiation (due to the log odds nature of the smoking initiation data). Sleep duration was measured in hours per day. Undersleeping was defined as sleeping ≤6 hours versus 7–8 hours and oversleeping as ≥9 hours versus 7–8 hours. Chronotype was measured on a five-point scale, with higher scores indicating being more of a “morning person” and lower scores more of an “evening person.” Insomnia was measured as usually having trouble falling asleep at night or waking up in the middle of the night (cases) versus never/rarely or sometimes having these problems (controls). CI = confidence interval; IVW = inverse-variance weighted fixed effects-meta-analysis; RAPS = Robust Adjusted Profile Score; SNP = single nucleotide polymorphism; OR = odds ratio.

In the other direction, there was no clear evidence for causal effects of sleep duration or chronotype on smoking behavior (Table 3). There was weak evidence for insomnia increasing the number of cigarettes smoked per day, but only with the genetic instrument combining 16 SNPs (IVW beta = 1.21, 95% CI = 0.20 to 2.22, *p* = .02). This was confirmed with both the weighted median regression (beta = 1.36, 95% CI = −0.10 to 2.81, *p* = .07) and MR-RAPS (beta = 1.26, 95% CI = 0.18 to 2.34, *p* = .02) sensitivity methods. Although MR-Egger regression showed no clear evidence for a causal effect (beta = −1.95, 95% CI = −6.79 to 2.89, *p* = .44), the Egger intercept indicated no directional pleiotropy (beta = .17, 95% CI = −0.08 to 0.41, *p* = .20; Supplementary Table 14), and so it is most likely that this analysis was underpowered. There was also weak evidence that insomnia causally influences smoking cessation such that having insomnia complaints decreases the odds of being a former versus a current smoker (IVW OR = 0.80, 95% CI = 0.65 to 0.97, *p* = .02). This was confirmed with MR-RAPS (OR = 0.79, 95% CI = 0.64 to 0.97, *p* = .02) and MR-Egger regression (OR = 0.31, 95% CI = 0.11 to 0.87, *p* = .04). The weighted median method showed no clear statistical evidence for a causal effect, but did confirm the direction of effect (OR = 0.86, 95% CI = 0.66 to 1.14, *p* = .30). There was some weak evidence for pleiotropy from the Egger intercept (OR = 1.05, 95% CI = 0.999 to 1.11, *p* = .09; Supplementary Table 14). For all other relationships tested, there was no clear evidence for causality.

**Table 3.** Two-sample Mendelian Randomisation (MR) Analyses with Summary-Level Data, Estimating Causal Effects of Sleep Behaviours (Sleep Duration, Chronotype and Insomnia) on Smoking Behaviors (Smoking Initiation, Cigarettes per Day, Smoking Cessation).

			N SNPs	Wald Ratio/ IVW		Weighted median		MR-RAPS		MR-Egger	
Exposure	Outcome	Threshold		Beta/OR (95% CI)	*p*	Beta/OR (95% CI)	*p*	Beta/OR (95% CI)	*p*	beta/OR (95% CI)	*p*
Sleep duration	Smoking initiation	*p*<5 × 10^−8^	3	1.12 (0.47 to 2.64)	0.81	1.01 (0.51 to 1.99)	0.97	—	—	—	—
		*p*<1 × 10^−5^	23	0.91 (0.67 to 1.25)	0.55	0.92 (0.63 to 1.35)	0.67	0.86 (0.62 to 1.18)	0.35	0.23 (0.04 to 1.42)	0.13
Sleep duration	Cigarettes per day	*p*<5 × 10^−8^	3	1.50 (−2.61 to 5.61)	0.47	1.08 (−3.36 to 5.52)	0.63	—	—	—	—
		*p*<1 × 10^−5^	23	0.59 (−1.19 to 2.37)	0.51	1.02 (−1.61 to 3.65)	0.45	0.62 (−1.23 to 2.47)	0.52	2.20 (−8.46 to 12.86)	0.69
Sleep duration	Smoking cessation	*p*<5 × 10^−8^	3	0.77 (0.37 to 1.58)	0.48	0.77 (0.32 to 1.82)	0.55	—	—	—	—
		*p*<1 × 10^−5^	23	0.75 (0.54 to 1.06)	0.10	0.81 (0.49 to 1.35)	0.42	0.74 (0.51 to 1.06)	0.10	0.83 (0.10 to 1.91)	0.87
Undersleeping	Smoking initiation	*p*<1 × 10^−5^	15	0.94 (0.40 to 2.18)	0.88	0.88 (0.31 to 2.51)	0.82	0.93 (0.43 to 0.72)	0.86	0.10 (0.00 to 58.78)	0.49
Undersleeping	Cigarettes per day	*p*<1 × 10^−5^	15	3.64 (−1.60 to 8.89)	0.17	3.38 (−3.92 to 10.69)	0.36	3.75 (−1.85 to 9.36)	0.19	14.80 (−15.53 to 45.14)	0.36
Undersleeping	Smoking cessation	*p*<1 × 10^−5^	15	1.21 (0.45 to 3.32)	0.71	1.10 (0.28 to 4.35)	0.90	1.22 (0.41 to 3.62)	0.72	0.58 (0.00 to 626.77)	0.88
Oversleeping	Smoking initiation	*p*<1 × 10^−5^	12	0.80 (0.27 to 2.39)	0.68	0.74 (0.16 to 3.35)	0.69	0.79 (0.25 to 0.92)	0.69	6.46 (1.00 to 41.63)	0.08
Oversleeping	Cigarettes per day	*p*<1 × 10^−5^	12	3.26 (−4.41 to 10.93)	0.40	4.56 (−5.34 to 14.47)	0.40	3.32 (−4.87 to 11.52)	0.43	2.91 (−7.94 to 13.76)	0.61
Oversleeping	Smoking cessation	*p*<1 × 10^−5^	12	0.58 (0.06 to 6.11)	0.65	1.10 (0.11 to 11.44)	0.94	0.55 (0.12 to 2.53)	0.44	0.003 (0.00 to 1.21)	0.09
Chronotype	Smoking initiation	*p*<5 × 10^−8^	8	0.78 (0.51 to 1.19)	0.24	0.75 (0.47 to 1.19)	0.22	—	—	—	—
		*p*<1 × 10^−5^	55	0.96 (0.81 to 1.13)	0.60	0.99 (0.79 to 1.25)	0.93	0.96 (0.80 to 1.14)	0.61	3.06 (1.96 to 4.78)	8 × 10^−6^
Chronotype	Cigarettes per day	*p*<5 × 10^−8^	8	−0.12 (−4.26 to 4.02)	0.96	−0.10 (−3.85 to 3.66)	0.96	—	—	—	—
		*p*<1 × 10^−5^	55	0.35 (−1.05 to 1.75)	0.62	−0.02 (−1.84 to 1.81)	0.99	0.36 (−1.10 to 1.81)	0.63	0.86 (−3.78 to 5.50)	0.72
Chronotype	Smoking cessation	*p*<5 × 10^−8^	8	1.01 (0.61 to 1.64)	0.99	1.08 (0.58 to 2.03)	0.80	—	—	—	—
		*p*<1 × 10^−5^	55	0.84 (0.66 to 1.06)	0.15	0.87 (0.62 to 1.23)	0.43	0.84 (0.66 to 1.07)	0.15	1.25 (0.61 to 2.57)	0.55
Insomnia	Smoking initiation	*p*<5 × 10^−8^	1	0.96 (0.55 to 1.67)	0.89	—	—	—	—	—	—
		*p*<1 × 10^−5^	16	0.92 (0.78 to 1.09)	0.40	1.03 (0.83 to 1.28)	0.79	0.93 (0.78 to 1.11)	0.43	1.04 (0.40 to 2.73)	0.94
Insomnia	Cigarettes per day	*p*<5 × 10^−8^	1	−1.56 (−5.78 to 2.66)	0.47	—	—	—	—	—	—
		*p*<1 × 10^−5^	16	1.21 (0.20 to 2.22)	0.02	1.36 (−0.10 to 2.81)	0.07	1.26 (0.18 to 2.34)	0.02	−1.95 (−6.79 to 2.89)	0.44
		*p*<1 × 10^−5^*	38	0.05 (−0.32 to 0.42)	0.80	−0.18 (−0.68 to 0.33)	0.50	0.05 (−0.33 to 0.43)	0.80	−0.04 (−0.80 to 0.73)	0.93
Insomnia	Smoking cessation	*p*<5 × 10^−8^	1	0.82 (0.34 to 1.95)	0.66	—	—	—	—	—	—
		*p*<1 × 10^−5^	16	0.80 (0.65 to 0.97)	0.02	0.86 (0.66 to 1.14)	0.30	0.79 (0.64 to 0.97)	0.02	0.31 (0.11 to 0.87)	0.04
		*p*<1 × 10^−5^*	38	0.91 (0.84 to 0.98)	0.02	0.94 (0.84 to 1.05)	0.30	0.91 (0.84 to 0.99)	0.04	1.22 (0.99 to 1.51)	0.07

Coefficients represent the change in outcome for a one-unit increase in the exposure variable in the case of continuous exposure variables and the change in outcome per 2.72-fold increase in the prevalence of the exposure for binary exposure variables (due to the log odds nature of the binary exposure data). Sleep duration was measured in hours per day. Undersleeping was defined as sleeping ≤6 hours versus 7-8 hours and oversleeping as ≥9 hours versus 7-8 hours. Chronotype was measured on a five-point scale, with higher scores indicating being more of a ‘morning person’ and lower scores more of an ‘evening person’. Insomnia was measured as usually having trouble falling asleep at night or waking up in the middle of the night (‘cases’) versus never/rarely or sometimes having these problems (‘controls’). Smoking initiation was measured as ever smoking versus never smoking. Smoking cessation as former smoking versus current smoking. * Replication with the 2^nd^ release of UK biobank as the outcome-sample (*n*=67,193 for cigarettes per day / *n*=107,874 for smoking cessation) instead of the TAG consortium as the outcome-sample (*n*=38,181 / *n*=67,193, respectively).

We attempted to replicate the aforementioned suggestive findings—implying causal effects of insomnia on cigarettes per day and smoking cessation—using the second release of UK Biobank as the outcome-sample. These analyses showed no clear evidence for a causal effect of insomnia on cigarettes smoked per day (IVW beta = .05, 95% CI = −0.32 to 0.42, *p* = .80) and confirmed weak evidence for a causal effect of insomnia on smoking cessation (IVW beta = .91, 95% CI = 0.84 to 0.98, *p* = .02; Table 3).

## Discussion

We found evidence of moderate genetic correlations between smoking and sleep behaviors. Genetic variants that were associated with insomnia also increased the odds of initiating smoking and were associated with smoking more cigarettes per day. Consistent with this finding, genetic variants that predicted shorter sleep duration increased the odds of initiating smoking and smoking more cigarettes per day. Finally, genetic variants that made it more likely to be a morning person (chronotype) were associated with a lower odds of smoking cessation. Possible causal relationships underlying these genetic correlations were tested with MR analyses. Using MR with individual-level data, we found compelling evidence that smoking more cigarettes per day decreases the odds of being a morning person. MR with summary-level data indicated weak evidence for insomnia causally increasing the number of cigarettes smoked and decreasing the odds of quitting smoking.

Although a genetic variant that is robustly associated with smoking heaviness was associated with a *lower* likelihood of being a morning person in smokers—consistent with a causal effect of smoking heaviness on chronotype—this same genetic variant was associated with a *higher* likelihood of morningness in never-smokers. This suggests that there is pleiotropy, such that the genetic instrument plays a (direct) role in chronotype. However, given that the direction of effect in never-smokers is opposite to that in current smokers, and in former smokers the effect is in the same direction as smokers but less strong, it is unlikely that pleiotropy is driving the association we see in smokers. This suggests that previously reported observational associations between smoking, nicotine dependence, and being an evening person^7^ may at least in part be explained by a causal effect of smoking on chronotype. One explanation for this is that the psychoactive properties of nicotine allow individuals to stay more alert into the evening.^7^ There is also evidence from animal research that both nicotine and nicotinic acetylcholine receptors (nAChRs) play a role in the circadian system.^22,33^ This could explain our finding of an association in never-smokers, given that the genetic variant, rs16969968, is a missense mutation that causes a functional change in the α5 nAChR subunit protein.^34^

MR with summary-level data provided suggestive evidence that insomnia causally increases smoking heaviness. Although the evidence was weak, and it was not replicated in a separate, larger outcome sample, it was supported by a considerable genetic correlation between insomnia and cigarettes per day (*r*g = .27). In line with this finding, there was weak evidence that insomnia decreases the odds of smoking cessation, which did replicate in a separate outcome sample. This is in accordance with observational evidence showing that pre-quit insomnia symptoms predict smoking cessation failure.^6,35,36^ One mechanism underlying a causal relationship from insomnia to smoking heaviness and cessation could be that smokers self-medicate against fatigue with cigarettes.^21^ If smoking cigarettes alleviates the negative consequences of insomnia, this could make it harder for smokers with insomnia to give up smoking. We did not find similar evidence for causal effects of sleep duration (in hours or as undersleeping) on smoking behavior, which may seem unexpected. However, insomnia is not necessarily characterized by a short *total* sleep duration^37^ and individuals with a naturally short duration of sleep may not experience fatigue. Our suggestive findings that insomnia causally increases smoking heaviness and decreases the odds of quitting can be informative for developing strategies to improve smoking cessation success. Treatments that are currently offered (nicotine-replacement therapies and medications such as bupropion or varenicline) are only moderately effective^38^ and insomnia may be a promising novel target that could complement existing treatments. This was also highlighted in a recent review article, which reported extensive conceptual support for sleep therapy as an adjunctive treatment for smoking.^39^

Recently, a study in UK Biobank participants (*n* = 498 208) reported observational associations between smoking and sleep behaviors, adjusting for sociodemographic variables, self-reported stress, depression, alcohol and coffee consumption, physical activity, shift work, and chronotype.^3^ Current smoking versus never smoking was associated with a higher odds of oversleeping (OR = 1.47, 95% CI = 1.17 to 1.85) whereas heavy current smoking (>20 cigarettes/day) versus never smoking was associated with a higher odds of both undersleeping (OR = 1.46, 95% CI = 1.15 to 1.87) and oversleeping (OR = 2.85, 95% CI = 1.66 to 4.89). Being a former versus a never-smoker was associated with experiencing more sleeplessness (OR = 1.10, 95% CI = 1.07 to 1.14).^3^ The authors did not investigate associations with chronotype, but instead adjusted for it in their main analyses. In this study, we did not find clear evidence for associations between smoking and oversleeping but we did confirm (genetic) associations between smoking and undersleeping. By also performing MR analyses, we added crucial knowledge on the possible causal nature of these associations, and their direction of effect.

Given the large sample sizes employed for our analyses, a major strength to our study is that we had much power to detect small effects. By combining multiple methods—LD-score regression, MR with individual-level data, and MR with summary-level data—we were able to differentiate shared genetic risk factors between smoking and sleep, from possible casual effects. A limitation to our study is that for some of the phenotypes (smoking initiation, undersleeping, oversleeping) we could only test effects with a genetic instrument that included SNPs under the threshold *p* less than 1 × 10^−5^. These instruments are less robustly associated with the exposure variable and therefore less precise when testing causal effects on an outcome variable. Another limitation is that our measures are based on self-report that could have introduced measurement error. Disrupted sleep may be a marker for general health, inducing false-positive findings when testing causal effects of smoking on sleep (given that smoking deteriorates health). However, it is unlikely that this has affected our results because we found that smoking influences chronotype but not sleep duration and/or insomnia. It should also be noted that the sleep duration measure includes daytime napping. Individuals who sleep little during the night but nap a lot during the day will seemingly have a healthy sleep duration. Although naps of ≤30 minutes are not a problem, longer naps can have negative health effects.^40^ However, there is no clear evidence that sleep disturbances and daytime napping are correlated,^41^ so it is unlikely that this has confounded our results. Finally, when interpreting our findings, the multiple testing burden should be taken into account, while also considering that some of the tests were not independent (eg, the variables undersleeping and sleep duration are based on the exact same data).

In summary, our findings suggest that smoking and sleep behaviors are genetically correlated and that some of this correlation reflects causal effects. Smoking more cigarettes per day seems to affect circadian rhythm (shifting it to being more of an “evening person”) whereas in the other direction we found weak evidence that insomnia increases smoking heaviness and decreases the odds of successfully quitting smoking. These findings increase our knowledge of the complex relationship between smoking and sleep and can help develop more evidence-based treatments for nicotine dependence by focusing on insomnia as a novel treatment target.

## Funding

MRM is a member of the UK Centre for Tobacco and Alcohol Studies, a UKCRC Public Health Research: Centre of Excellence. Funding from British Heart Foundation, Cancer Research UK, Economic and Social Research Council, Medical Research Council, and National Institute for Health Research, under the auspices of the UK Clinical Research Collaboration, is gratefully acknowledged. This work was supported by the Medical Research Council Integrative Epidemiology Unit at the University of Bristol, which is supported by the Medical Research Council and the University of Bristol (grants MC_UU_12013/6 and MC_UU_12013/7). JLT is supported by a Rubicon grant from the Netherlands Organization for Scientific Research (NWO; grant number 446-16-009).

## Declaration of Interests

*None declared.*

## Supplementary Material

nty230_suppl_Supplementary_Material
