# Supplementary material for: Evidence for Genetic Correlations and Bidirectional, Causal Effects Between Smoking and Sleep Behaviors
Source: Nicotine Tob Res. 2018 Oct 26;21(6):731–8. doi: 10.1093/ntr/nty230 (PMC6528151; doi:10.1093/ntr/nty230)
Supplement: nty230_suppl_Supplementary_Material [file nty230_suppl_supplementary_material.docx]

**Supplementary Material**

*‘Evidence for genetic correlations and bidirectional, causal effects between smoking and sleep behaviours’*

Mark Gibson, Marcus R Munafò, Amy E Taylor, Jorien L Treur

**Content:**

Page 2-4 Supplementary Methods.

Page 5 Supplementary Table 1. Associations of rs16969968 with smoking heaviness in UK Biobank

Page 6 Supplementary Table 2. Associations of rs3025343 with smoking cessation in UK Biobank

Page 7 Supplementary Table 3. Associations of rs16969968 / rs3025343 with potential confounders (in the full sample)

Page 8 Supplementary Table 4. Associations of rs16969968 / rs3025343 with potential confounders (in never smokers only)

Page 9 Supplementary Table 5. Associations of rs16969968 / rs3025343 with potential confounders (in former smokers only)

Page 10 Supplementary Table 6. Associations of rs16969968 / rs3025343 with potential confounders (in current smokers only)

Page 11 Supplementary Table 7. Associations of rs16969968 / rs3025343 with potential confounders (in ever smokers only)

Page 12-36 Supplementary Table 8. List of SNPs included in the genetic instruments used for MR

Page 37 Supplementary Table 9. Cochran's Heterogeneity statistic for Inverse Variance Weighted (IVW) two-sample MR from smoking initiation to sleep behaviours

Page 38 Supplementary Table 10. I-squared statistic for two-sample MR from smoking initiation to sleep behaviours

Page 39 Supplementary Table 11. MR-Egger intercept for two-sample MR from smoking initiation to sleep behaviours

Page 40 Supplementary Table 12. Cochran's Heterogeneity statistic for Inverse Variance Weighted (IVW) two-sample MR from sleep behaviours to smoking behaviours

Page 41 Supplementary Table 13. I-squared statistic for two-sample MR from sleep behaviours to smoking behaviours

Page 42 Supplementary Table 14. MR-Egger intercept for two-sample MR from sleep behaviours to smoking behaviours

**Supplementary Methods**

UK Biobank is a population-based health research resource consisting of approximately 500,000 people, aged between 38 years and 73 years, who were recruited between the years 2006 and 2010 from across the UK (1) . Particularly focused on identifying determinants of human diseases in middle-aged and older individuals, participants provided a range of information (such as demographics, health status, lifestyle measures, cognitive testing, personality self-report, and physical and mental health measures) via questionnaires and interviews; anthropometric measures, BP readings and samples of blood, urine and saliva were also taken (data available at www.ukbiobank.ac.uk). A full description of the study design, participants and quality control (QC) methods have been described in detail previously (2). UK Biobank received ethical approval from the Research Ethics Committee (REC reference for UK Biobank is 11/NW/0382).

**Smoking status**

Participants were asked about current and past tobacco (cigarette, pipe, cigar or other) smoking behaviour in a computerized questionnaire. A full list of the questions is available at: <http://biobank.ctsu.ox.ac.uk/crystal/docs/TouchscreenQuestionsMainFinal.pdf>. The following questions were asked about current and past smoking status: “Do you smoke tobacco now?” (Yes, on most or all days, Only occasionally, No, Prefer not to answer) and “In the past, how often have you smoked tobacco?” (Smoked on most or all days, Smoked occasionally, Just tried once or twice, I have never smoked, Prefer not to answer). Individuals who indicated that they had tried tobacco but were not past or current daily or near daily smokers were also asked “Have you smoked more than 100 cigarettes in your lifetime?”. From these questions, the following smoking status categories were defined: never smokers (individuals who had smoked less than 100 cigarettes in a lifetime), former smokers (who had smoked more than 100 cigarettes but were not current smokers) and current smokers. Where individuals did not know or did not say whether they had consumed more than 100 cigarettes in their lifetime, those who said they had tried one or two in the past were classified as never smokers and those who said they smoked occasionally in the past as former smokers). Current regular smokers were asked about number of cigarettes consumed per day; answers were provided on a continuous scale. Pack years were calculated from reported daily cigarette consumption, age of starting smoking and age of stopping smoking (in former cigarette smokers) using the formula: pack years = (daily cigarette consumption/20) × years smoking. Years smoking was calculated as current age - age of starting in current smokers, and age of stopping - age of starting in former smokers. Where individuals reported starting smoking prior to age 16, pack years was calculated from age 16, as has been done in previous analyses of UK Biobank data (3). Where individuals reported starting and stopping smoking at the same age, it was assumed that they had smoked for 0.5 years.

**Sleep duration, insomnia and chronotype**

Participants were asked “About how many hours of sleep do you get in every 24 hours?” in complete hours. Those responding, “Do not know” or “Prefer not to answer” were coded as missing. Undersleeping and oversleeping were derived from sleep duration, with undersleeping being a binary variable indicating sleeping 6 or fewer hours per night compared to 7/8 and oversleeping a binary variable indicating 9 or more hours per night compared to 7/8.

The question about insomnia was “Do you have trouble falling asleep at night or do you wake up in the middle of the night?” Participants answering “Never/rarely” or “sometimes” were coded as 0, whilst those answering “usually” were coded as 1. Those responding “Prefer not to answer” were coded as missing. For chronotype, participants were asked “Do you consider yourself to be: “Definitely a morning person, More a morning person than an evening person, Don’t know, More an evening than a morning person, Definitely an evening person”? Answers were coded from -2 to 2, with -2 being a definite evening person and 2 a definite morning person. Those responding “Prefer not to answer” were coded as missing.

**Genotyping and imputation**

The full data release contains the cohort of successfully genotyped samples (n=488,377). 49,979 individuals were genotyped using the UK BiLEVE array and 438,398 using the UK Biobank axiom array. Pre-imputation QC, phasing and imputation are described elsewhere (4). In brief, prior to phasing, multiallelic SNPs or those with MAF ≤1% were removed. Phasing of genotype data was performed using a modified version of the SHAPEIT2 algorithm (5). Genotype imputation to a reference set combining the UK10K haplotype and HRC reference panels (6) was performed using IMPUTE2 algorithms (7) . The analyses presented here were restricted to autosomal variants within the HRC site list using a graded filtering with varying imputation quality for different allele frequency ranges. Therefore, rarer genetic variants are required to have a higher imputation INFO score (Info>0.3 for MAF >3%; Info>0.6 for MAF 1-3%; Info>0.8 for MAF 0.5-1%; Info>0.9 for MAF 0.1- 0.5%) with MAF and Info scores having been recalculated on an in house derived ‘European’ subset. Both rs16969968 and rs3025343 were imputed with an info score of 1 and did not show clear evidence of deviation from hardy Weinberg equilibrium (p>0.6).

**Data quality control**

Individuals with sex-mismatch (derived by comparing genetic sex and reported sex) or individuals with sex-chromosome aneuploidy were excluded from the analysis (n=814).

**Ancestry**

We restricted the sample to individuals of white British ancestry who self-report as “White British” and who have very similar ancestral backgrounds according to the PCA (n=409,703), as described by Bycroft (4).

**Degree of relatedness**

Estimated kinship coefficients using the KING toolset (8) identified 107,162 pairs of individuals (4). An in-house algorithm was then applied to this list and preferentially removed the individuals related to the greatest number of other individuals until no related pairs remain. These individuals were excluded (n=79,448). Additionally, 2 individuals were removed due to them relating to a very large number (>200) of individuals.

**References**

1. Allen NE, Sudlow C, Peakman T, Collins R, Biobank UK. UK biobank data: come and get it. Sci Transl Med. 2014;6(224):224ed4.

2. Collins R. What makes UK Biobank special? Lancet. 2012;379(9822):1173-4.

3. Wain LV, Shrine N, Miller S, Jackson VE, Ntalla I, Artigas MS, et al. Novel insights into the genetics of smoking behaviour, lung function, and chronic obstructive pulmonary disease (UK BiLEVE): a genetic association study in UK Biobank. Lancet Respir Med. 2015;3(10):769-81.

4. Bycroft C, Freeman C, Petkova D, Band G, Elliott LT, Sharp K, et al. Genome-wide genetic data on ~500,000 UK Biobank participants. BioRxiv. 2017.

5. O'Connell J, Sharp K, Shrine N, Wain L, Hall I, Tobin M, et al. Haplotype estimation for biobank-scale data sets. Nature genetics. 2016;48(7):817-20.

6. Huang J, Howie B, McCarthy S, Memari Y, Walter K, Min JL, et al. Improved imputation of low-frequency and rare variants using the UK10K haplotype reference panel. Nat Commun. 2015;6:8111.

7. Howie B, Marchini J, Stephens M. Genotype imputation with thousands of genomes. G3 (Bethesda). 2011;1(6):457-70.

8. Manichaikul A, Mychaleckyj JC, Rich SS, Daly K, Sale M, Chen WM. Robust relationship inference in genome-wide association studies. Bioinformatics. 2010;26(22):2867-73.

**Supplementary Table 1.** Associations of rs16969968 with smoking heaviness in UK Biobank

|  | N | Beta (95% CI) | p-value |
| --- | --- | --- | --- |
| Cigarettes per day in current smokers | 22,562 | 0.98 (0.82, 1.14) | 9.4E-34 |
| Pack years in ever smokers | 100,321 | 1.51 (1.34, 1.67) | 2.4E-74 |

Associations are per additional minor allele. Restricted to individuals reporting current daily or past regular smoking. Adjusted for age, sex and the first 4 genetic principal components.

**Supplementary Table 2.** Association of rs3025343 with smoking cessation in UK Biobank

|  | N | OR (95% CI) | p-value |
| --- | --- | --- | --- |
| Former vs current smoker | 151,621 | 1.12 (1.09, 1.15) | 6.7E-16 |

OR per additional minor allele. Adjusted for age, sex and the first 4 genetic principal components.

**Supplementary Table 3.** Associations with potential confounders (in the full sample)

|  |  | rs16969968^1^ | | rs3025343^2^ | |
| --- | --- | --- | --- | --- | --- |
|  | N | Beta/OR (95% CI) | p-value | Beta/OR (95% CI) | p-value |
| Sex (male vs female) | 337,106 | 0.99 (0.98, 1.00) | 0.21 | 1.00 (0.99. 1/02) | 0.78 |
| Age | 337,106 | -0.03 (-0.07, 0.01) | 0.20 | 0.04 (-0.02, 0.10) | 0.20 |
| Townsend deprivation index | 336,708 | 0.008 (-0.007, 0.022) | 0.32 | -0.003 (-0.03, 0.02) | 0.76 |
| Household income (5 categories) | 290,524 | -0.003 (-0.010, 0.003) | 0.34 | 0.002 (-0.007, 0.012) | 0.64 |
| Highest educational qualification (degree/professional vs other) | 333,977 | 1.00 (0.99, 1.01) | 0.80 | 0.98 (0.97, 0.99) | 0.04 |
| Alcohol consumption (daily vs non-daily) | 336,871 | 1.00 (0.99, 1.01) | 0.79 | 1.00 (0.98, 1.02) | 0.87 |

Higher Townsend deprivation index = more deprived. Household income categories: 1) <£18,000, 2) £18,000-30,999, 3) £31,000- 51,999, 4) £52,000-100,000, >£100,000

**Supplementary Table 4.** Associations with potential confounders (in never smokers only)

|  |  | rs16969968 | | rs3025343 | |
| --- | --- | --- | --- | --- | --- |
|  | N | Beta/OR (95% CI) | p-value | Beta/OR (95% CI) | p-value |
| Sex (male vs female) | 184,300 | 1.00 (0.98, 1.01) | 0.49 | 1.02 (0.99, 1.04) | 0.15 |
| Age | 184,300 | 0.06 (0.002, 0.11) | 0.04 | 0.09 (0.01, 0.17) | 0.03 |
| Townsend deprivation index | 184,073 | 0.01, (-0.006, 0.031) | 0.19 | -0.009, -0.037, 0.018) | 0.50 |
| Household income (5 categories) | 158,124 | -0.004 (-0.013, 0.004) | 0.33 | 0.006 (-0.007, 0.019) | 0.39 |
| Highest educational qualification (degree/professional vs other) | 182,701 | 1.00 (0.98. 1.01) | 0.73 | 0.99 (0.97, 1.01) | 0.48 |
| Alcohol consumption (daily vs non-daily) | 184,203 | 0.99 (0.97, 1.01) | 0.22 | 1.00 (0.97, 1.02) | 0.81 |

Higher Townsend deprivation index = more deprived. Household income categories: 1) <£18,000, 2) £18,000-30,999, 3) £31,000- 51,999, 4) £52,000-100,000, >£100,000

**Supplementary Table 5.** Associations with potential confounders (in former smokers only)

|  |  | rs16969968 | |
| --- | --- | --- | --- |
|  | N | Beta/OR (95% CI) | p-value |
| Sex (male vs female) | 118,257 | 1.00 (0.98, 1.02) | 0.90 |
| Age | 118,257 | -0.09 (-0.15, 0.02) | 0.009 |
| Townsend deprivation index | 118,132 | 0.0003 (-0.02, 0.03) | 0.98 |
| Household income (5 categories) | 102,691 | 0.001 (-0.009, 0.012) | 0.79 |
| Highest educational qualification (degree/professional vs other) | 117,190 | 1.01 (0.99, 1.02) | 0.57 |
| Alcohol consumption (daily vs non-daily) | 118,197 | 1.02 (1.00, 1.04) | 0.06 |

Higher Townsend deprivation index = more deprived. Household income categories: 1) <£18,000, 2) £18,000-30,999, 3) £31,000- 51,999, 4) £52,000-100,000, >£100,000

**Supplementary Table 6.** Associations with potential confounders (in current smokers only)

|  |  | rs16969968 | |
| --- | --- | --- | --- |
|  | N | Beta/OR (95% CI) | p-value |
| Sex (male vs female) | 33,364 | 0.99 (0.96, 1.02) | 0.58 |
| Age | 33,364 | -0.14 (-0.27, -0.004) | 0.04 |
| Townsend deprivation index | 33,320 | 0.02 (-0.03, 0.08) | 0.45 |
| Household income (5 categories) | 28,969 | -0.02 (-0.04, -0.002) | 0.03 |
| Highest educational qualification (degree/professional vs other) | 32,989 | 0.99 (0.96, 1.02) | 0.66 |
| Alcohol consumption (daily vs non-daily) | 33,310 | 0.99 (0.96, 1.03) | 0.65 |

Higher Townsend deprivation index = more deprived. Household income categories: 1) <£18,000, 2) £18,000-30,999, 3) £31,000- 51,999, 4) £52,000-100,000, >£100,000

**Supplementary Table 7.** Associations with potential confounders (in ever smokers only)

|  |  | rs3025343 | |
| --- | --- | --- | --- |
|  | N | beta/OR (95% CI) | p-value |
| Sex (male vs female) | 151,621 | 0.99 (0.97, 1.01) | 0.45 |
| Age | 151,621 | -0.01 (-0.10, -0.07) | 0.78 |
| Townsend deprivation index | 151,452 | 0.01 (-0.03, 0.04) | 0.71 |
| Household income (5 categories) | 131,660 | -0.003 (-0.02, 0.011) | 0.72 |
| Highest educational qualification (degree/professional vs other) | 150,179 | 0.97 (0.95, 0.99) | 0.01 |
| Alcohol consumption (daily vs non-daily) | 151,507 | 1.00 (0.98, 1.03) | 0.76 |

Higher Townsend deprivation index = more deprived. Household income categories: 1) <£18,000, 2) £18,000-30,999, 3) £31,000- 51,999, 4) £52,000-100,000, >£100,000

**Supplementary Table 8.** Single nucleotide polymorphisms (SNPs) included in the genetic instruments used for bidirectional, two-sample Mendelian randomization analyses between smoking and sleep behaviours.

|  |  |  |  |  |  |  | Gene-exposure estimate | | | Gene-outcome estimate | | |
| --- | --- | --- | --- | --- | --- | --- | --- | --- | --- | --- | --- | --- |
| Exposure | Outcome | Threshold genetic instrument | SNP | Original SNP if proxy used (LD R^2^ with original SNP) | Effect allele | Effect allele frequency | beta | SE | p-value | beta | SE | p-value |
| Smoking initiation | Sleep duration | *p*<1×10^-5^ | rs10013579 |  | T | 0.627 | 0.058 | 0.013 | 3.5E-06 | -0.001 | 0.004 | 0.84 |
|  |  |  | rs10108954 |  | T | 0.067 | -0.167 | 0.035 | 1.6E-06 | 0.003 | 0.012 | 0.83 |
|  |  |  | rs10937751 |  | A | 0.300 | 0.066 | 0.014 | 2.8E-06 | -0.003 | 0.005 | 0.57 |
|  |  |  | rs11030084 |  | T | 0.209 | -0.067 | 0.015 | 7.2E-06 | 0.000 | 0.005 | 0.95 |
|  |  |  | rs11067275 |  | T | 0.669 | 0.065 | 0.014 | 2.7E-06 | 0.000 | 0.004 | 0.94 |
|  |  |  | rs11892348 |  | A | 0.346 | -0.054 | 0.012 | 8.6E-06 | 0.008 | 0.004 | 0.04 |
|  |  |  | rs13131292 |  | A | 0.190 | -0.107 | 0.024 | 7.6E-06 | -0.008 | 0.006 | 0.17 |
|  |  |  | rs16904189 |  | T | 0.923 | -0.152 | 0.034 | 7.5E-06 | -0.002 | 0.011 | 0.86 |
|  |  |  | rs16941640 |  | A | 0.116 | 0.130 | 0.025 | 2.2E-07 | 0.001 | 0.008 | 0.85 |
|  |  |  | rs1839129 |  | C | 0.285 | -0.063 | 0.014 | 5.7E-06 | 0.004 | 0.005 | 0.41 |
|  |  |  | rs1986692 |  | A | 0.602 | 0.058 | 0.013 | 7.4E-06 | -0.005 | 0.004 | 0.22 |
|  |  |  | rs241526 |  | T | 0.482 | -0.053 | 0.012 | 8.6E-06 | -0.002 | 0.004 | 0.68 |
|  |  |  | rs2449222 |  | T | 0.881 | -0.091 | 0.020 | 5.3E-06 | -0.008 | 0.007 | 0.24 |
|  |  |  | rs2742680 |  | C | 0.195 | -0.099 | 0.021 | 2.3E-06 | 0.010 | 0.005 | 0.06 |
|  |  |  | rs3782288 |  | T | 0.834 | 0.085 | 0.018 | 4.0E-06 | 0.008 | 0.006 | 0.23 |
|  |  |  | rs725695 |  | A | 0.407 | -0.055 | 0.012 | 5.0E-06 | 0.000 | 0.004 | 0.97 |
|  |  |  | rs739484 |  | T | 0.142 | -0.088 | 0.020 | 9.0E-06 | 0.009 | 0.007 | 0.19 |
|  |  |  | rs7548367 |  | C | 0.660 | 0.056 | 0.013 | 9.0E-06 | 0.001 | 0.004 | 0.74 |
|  |  |  | rs9521281 |  | T | 0.200 | -0.069 | 0.015 | 8.4E-06 | 0.006 | 0.005 | 0.27 |
| Smoking initiation | Undersleeping | *p*<1×10^-5^ | rs10013579 |  | T | 0.627 | 0.058 | 0.013 | 3.5E-06 | -0.002 | 0.002 | 0.39 |
|  |  |  | rs10108954 |  | T | 0.067 | -0.167 | 0.035 | 1.6E-06 | 0.000 | 0.006 | 0.97 |
|  |  |  |  |  |  |  | Gene-exposure estimate | | | Gene-outcome estimate | | |
| Exposure | Outcome | Threshold genetic instrument | SNP | Original SNP if proxy used (LD R^2^ with original SNP) | Effect allele | Effect allele frequency | beta | SE | p-value | beta | SE | p-value |
|  |  |  | rs10937751 |  | A | 0.300 | 0.066 | 0.014 | 2.8E-06 | 0.001 | 0.002 | 0.60 |
|  |  |  | rs11030084 |  | T | 0.209 | -0.067 | 0.015 | 7.2E-06 | 0.001 | 0.002 | 0.76 |
|  |  |  | rs11067275 |  | T | 0.669 | 0.065 | 0.014 | 2.7E-06 | 0.002 | 0.002 | 0.37 |
|  |  |  | rs11892348 |  | A | 0.346 | -0.054 | 0.012 | 8.6E-06 | -0.003 | 0.002 | 0.16 |
|  |  |  | rs13131292 |  | A | 0.190 | -0.107 | 0.024 | 7.6E-06 | 0.001 | 0.003 | 0.68 |
|  |  |  | rs16904189 |  | T | 0.923 | -0.152 | 0.034 | 7.5E-06 | -0.001 | 0.005 | 0.90 |
|  |  |  | rs16941640 |  | A | 0.116 | 0.130 | 0.025 | 2.2E-07 | 0.002 | 0.003 | 0.58 |
|  |  |  | rs1839129 |  | C | 0.285 | -0.063 | 0.014 | 5.7E-06 | -0.003 | 0.002 | 0.15 |
|  |  |  | rs1986692 |  | A | 0.602 | 0.058 | 0.013 | 7.4E-06 | 0.003 | 0.002 | 0.12 |
|  |  |  | rs241526 |  | T | 0.482 | -0.053 | 0.012 | 8.6E-06 | 0.001 | 0.002 | 0.69 |
|  |  |  | rs2449222 |  | T | 0.881 | -0.091 | 0.020 | 5.3E-06 | 0.000 | 0.003 | 0.91 |
|  |  |  | rs2742680 |  | C | 0.195 | -0.099 | 0.021 | 2.3E-06 | -0.003 | 0.002 | 0.16 |
|  |  |  | rs3782288 |  | T | 0.834 | 0.085 | 0.018 | 4.0E-06 | 0.000 | 0.003 | 0.91 |
|  |  |  | rs725695 |  | A | 0.407 | -0.055 | 0.012 | 5.0E-06 | 0.000 | 0.002 | 0.90 |
|  |  |  | rs739484 |  | T | 0.142 | -0.088 | 0.020 | 9.0E-06 | -0.003 | 0.003 | 0.29 |
|  |  |  | rs7548367 |  | C | 0.660 | 0.056 | 0.013 | 9.0E-06 | -0.003 | 0.002 | 0.18 |
|  |  |  | rs9521281 |  | T | 0.200 | -0.069 | 0.015 | 8.4E-06 | -0.002 | 0.002 | 0.33 |
| Smoking initiation | Oversleeping | *p*<1×10^-5^ | rs10013579 |  | T | 0.627 | 0.058 | 0.013 | 3.5E-06 | -0.002 | 0.001 | 0.25 |
|  |  |  | rs10108954 |  | T | 0.067 | -0.167 | 0.035 | 1.6E-06 | 0.002 | 0.004 | 0.64 |
|  |  |  | rs10937751 |  | A | 0.300 | 0.066 | 0.014 | 2.8E-06 | 0.001 | 0.002 | 0.56 |
|  |  |  | rs11030084 |  | T | 0.209 | -0.067 | 0.015 | 7.2E-06 | 0.002 | 0.002 | 0.33 |
|  |  |  | rs11067275 |  | T | 0.669 | 0.065 | 0.014 | 2.7E-06 | 0.001 | 0.002 | 0.62 |
|  |  |  | rs11892348 |  | A | 0.346 | -0.054 | 0.012 | 8.6E-06 | 0.001 | 0.001 | 0.56 |
|  |  |  |  |  |  |  | Gene-exposure estimate | | | Gene-outcome estimate | | |
| Exposure | Outcome | Threshold genetic instrument | SNP | Original SNP if proxy used (LD R^2^ with original SNP) | Effect allele | Effect allele frequency | beta | SE | p-value | beta | SE | p-value |
|  |  |  | rs13131292 |  | A | 0.190 | -0.107 | 0.024 | 7.6E-06 | -0.002 | 0.002 | 0.32 |
|  |  |  | rs16904189 |  | T | 0.923 | -0.152 | 0.034 | 7.5E-06 | -0.001 | 0.004 | 0.87 |
|  |  |  | rs16941640 |  | A | 0.116 | 0.130 | 0.025 | 2.2E-07 | 0.004 | 0.003 | 0.19 |
|  |  |  | rs1839129 |  | C | 0.285 | -0.063 | 0.014 | 5.7E-06 | -0.001 | 0.002 | 0.56 |
|  |  |  | rs1986692 |  | A | 0.602 | 0.058 | 0.013 | 7.4E-06 | 0.001 | 0.001 | 0.68 |
|  |  |  | rs241526 |  | T | 0.482 | -0.053 | 0.012 | 8.6E-06 | 0.001 | 0.001 | 0.42 |
|  |  |  | rs2449222 |  | T | 0.881 | -0.091 | 0.020 | 5.3E-06 | 0.001 | 0.002 | 0.81 |
|  |  |  | rs2742680 |  | C | 0.195 | -0.099 | 0.021 | 2.3E-06 | 0.001 | 0.002 | 0.46 |
|  |  |  | rs3782288 |  | T | 0.834 | 0.085 | 0.018 | 4.0E-06 | 0.003 | 0.002 | 0.24 |
|  |  |  | rs725695 |  | A | 0.407 | -0.055 | 0.012 | 5.0E-06 | 0.002 | 0.001 | 0.29 |
|  |  |  | rs739484 |  | T | 0.142 | -0.088 | 0.020 | 9.0E-06 | 0.002 | 0.002 | 0.42 |
|  |  |  | rs7548367 |  | C | 0.660 | 0.056 | 0.013 | 9.0E-06 | 0.001 | 0.001 | 0.62 |
|  |  |  | rs9521281 |  | T | 0.200 | -0.069 | 0.015 | 8.4E-06 | -0.001 | 0.002 | 0.61 |
| Smoking initiation | Chronotype | *p*<1×10^-5^ | rs10013579 |  | T | 0.627 | 0.058 | 0.013 | 3.5E-06 | -0.002 | 0.004 | 0.56 |
|  |  |  | rs10108954 |  | T | 0.067 | -0.167 | 0.035 | 1.6E-06 | 0.003 | 0.012 | 0.77 |
|  |  |  | rs10937751 |  | A | 0.300 | 0.066 | 0.014 | 2.8E-06 | 0.000 | 0.005 | 0.95 |
|  |  |  | rs11030084 |  | T | 0.209 | -0.067 | 0.015 | 7.2E-06 | 0.015 | 0.005 | 0.003 |
|  |  |  | rs11067275 |  | T | 0.669 | 0.065 | 0.014 | 2.7E-06 | -0.003 | 0.004 | 0.42 |
|  |  |  | rs11892348 |  | A | 0.346 | -0.054 | 0.012 | 8.6E-06 | 0.005 | 0.004 | 0.26 |
|  |  |  | rs13131292 |  | A | 0.190 | -0.107 | 0.024 | 7.6E-06 | 0.002 | 0.006 | 0.70 |
|  |  |  | rs16904189 |  | T | 0.923 | -0.152 | 0.034 | 7.5E-06 | 0.021 | 0.010 | 0.04 |
|  |  |  | rs16941640 |  | A | 0.116 | 0.130 | 0.025 | 2.2E-07 | 0.006 | 0.008 | 0.43 |
|  |  |  | rs1817648 |  | T | 0.467 | -0.052 | 0.012 | 7.8E-06 | -0.015 | 0.004 | 8.70E-05 |
|  |  |  |  |  |  |  | Gene-exposure estimate | | | Gene-outcome estimate | | |
| Exposure | Outcome | Threshold genetic instrument | SNP | Original SNP if proxy used (LD R^2^ with original SNP) | Effect allele | Effect allele frequency | beta | SE | p-value | beta | SE | p-value |
|  |  |  | rs1839129 |  | C | 0.285 | -0.063 | 0.014 | 5.7E-06 | 0.003 | 0.005 | 0.50 |
|  |  |  | rs1986692 |  | A | 0.602 | 0.058 | 0.013 | 7.4E-06 | 0.005 | 0.004 | 0.19 |
|  |  |  | rs241526 |  | T | 0.482 | -0.053 | 0.012 | 8.6E-06 | -0.001 | 0.004 | 0.90 |
|  |  |  | rs2449222 |  | T | 0.881 | -0.091 | 0.020 | 5.3E-06 | -0.009 | 0.007 | 0.19 |
|  |  |  | rs2742680 |  | C | 0.195 | -0.099 | 0.021 | 2.3E-06 | 0.001 | 0.005 | 0.84 |
|  |  |  | rs3782288 |  | T | 0.834 | 0.085 | 0.018 | 4.0E-06 | 0.007 | 0.006 | 0.27 |
|  |  |  | rs725695 |  | A | 0.407 | -0.055 | 0.012 | 5.0E-06 | -0.004 | 0.004 | 0.33 |
|  |  |  | rs739484 |  | T | 0.142 | -0.088 | 0.020 | 9.0E-06 | -0.004 | 0.006 | 0.51 |
|  |  |  | rs7548367 |  | C | 0.660 | 0.056 | 0.013 | 9.0E-06 | -0.001 | 0.004 | 0.73 |
|  |  |  | rs9521281 |  | T | 0.200 | -0.069 | 0.015 | 8.4E-06 | 0.002 | 0.005 | 0.72 |
| Smoking initiation | Insomnia | *p*<1×10^-5^ | rs10013579 |  | T | 0.627 | 0.058 | 0.013 | 3.5E-06 | -0.014 | 0.010 | 0.15 |
|  |  |  | rs10108954 |  | T | 0.067 | -0.167 | 0.035 | 1.6E-06 | 0.001 | 0.028 | 0.96 |
|  |  |  | rs10937751 |  | A | 0.300 | 0.066 | 0.014 | 2.8E-06 | 0.001 | 0.011 | 0.95 |
|  |  |  | rs11030084 |  | T | 0.209 | -0.067 | 0.015 | 7.2E-06 | 0.019 | 0.012 | 0.12 |
|  |  |  | rs11067275 |  | T | 0.669 | 0.065 | 0.014 | 2.7E-06 | 0.004 | 0.010 | 0.67 |
|  |  |  | rs11892348 |  | A | 0.346 | -0.054 | 0.012 | 8.6E-06 | 0.001 | 0.010 | 0.89 |
|  |  |  | rs13131292 |  | A | 0.190 | -0.107 | 0.024 | 7.6E-06 | -0.012 | 0.014 | 0.39 |
|  |  |  | rs16904189 |  | T | 0.923 | -0.152 | 0.034 | 7.5E-06 | -0.016 | 0.025 | 0.51 |
|  |  |  | rs16941640 |  | A | 0.116 | 0.130 | 0.025 | 2.2E-07 | 0.025 | 0.018 | 0.17 |
|  |  |  | rs1817648 |  | T | 0.467 | -0.052 | 0.012 | 7.8E-06 | -0.001 | 0.009 | 0.92 |
|  |  |  | rs1839129 |  | C | 0.285 | -0.063 | 0.014 | 5.7E-06 | -0.025 | 0.011 | 0.02 |
|  |  |  | rs1986692 |  | A | 0.602 | 0.058 | 0.013 | 7.4E-06 | 0.005 | 0.010 | 0.59 |
|  |  |  | rs241526 |  | T | 0.482 | -0.053 | 0.012 | 8.6E-06 | 0.008 | 0.010 | 0.43 |
|  |  |  |  |  |  |  | Gene-exposure estimate | | | Gene-outcome estimate | | |
| Exposure | Outcome | Threshold genetic instrument | SNP | Original SNP if proxy used (LD R^2^ with original SNP) | Effect allele | Effect allele frequency | beta | SE | p-value | beta | SE | p-value |
|  |  |  | rs2449222 |  | T | 0.881 | -0.091 | 0.020 | 5.3E-06 | 0.015 | 0.016 | 0.36 |
|  |  |  | rs2742680 |  | C | 0.195 | -0.099 | 0.021 | 2.3E-06 | -0.001 | 0.013 | 0.92 |
|  |  |  | rs3782288 |  | T | 0.834 | 0.085 | 0.018 | 4.0E-06 | 0.024 | 0.015 | 0.11 |
|  |  |  | rs725695 |  | A | 0.407 | -0.055 | 0.012 | 5.0E-06 | 0.011 | 0.010 | 0.23 |
|  |  |  | rs739484 |  | T | 0.142 | -0.088 | 0.020 | 9.0E-06 | 0.019 | 0.015 | 0.20 |
|  |  |  | rs7548367 |  | C | 0.660 | 0.056 | 0.013 | 9.0E-06 | -0.010 | 0.010 | 0.30 |
|  |  |  | rs9521281 |  | T | 0.200 | -0.069 | 0.015 | 8.4E-06 | 0.007 | 0.012 | 0.56 |
| Sleep duration | Smoking initiation | *p*<5×10^-8^ | rs1380703 |  | A | 0.618 | 0.025 | 0.004 | 7.6E-09 | 0.026 | 0.013 | 0.04 |
|  |  |  | rs17190618 |  | A | 0.840 | -0.033 | 0.005 | 1.2E-09 | 0.022 | 0.016 | 0.18 |
|  |  |  | rs1807282 | rs62158211 (0.99) | A | 0.788 | -0.039 | 0.005 | 8.7E-16 | -0.002 | 0.014 | 0.90 |
| Sleep duration | Smoking initiation | *p*<1×10^-5^ | rs10510128 |  | G | 0.795 | -0.022 | 0.005 | 4.6E-06 | 0.032 | 0.014 | 0.02 |
|  |  |  | rs10840160 |  | T | 0.536 | 0.019 | 0.004 | 2.7E-06 | 0.022 | 0.012 | 0.07 |
|  |  |  | rs11152363 |  | G | 0.813 | 0.023 | 0.005 | 8.3E-06 | -0.003 | 0.015 | 0.84 |
|  |  |  | rs11964802 |  | A | 0.712 | 0.019 | 0.004 | 8.5E-06 | -0.021 | 0.024 | 0.37 |
|  |  |  | rs1204056 |  | G | 0.338 | 0.015 | 0.004 | 3.0E-04 | 0.002 | 0.013 | 0.87 |
|  |  |  | rs12984777 |  | C | 0.972 | -0.058 | 0.012 | 2.1E-06 | 0.019 | 0.092 | 0.84 |
|  |  |  | rs1380703 |  | A | 0.618 | 0.025 | 0.004 | 7.6E-09 | 0.026 | 0.013 | 0.04 |
|  |  |  | rs16865859 |  | T | 0.869 | -0.027 | 0.006 | 3.2E-06 | 0.030 | 0.017 | 0.08 |
|  |  |  | rs16949934 |  | G | 0.920 | -0.034 | 0.007 | 3.3E-06 | 0.008 | 0.021 | 0.70 |
|  |  |  | rs17121264 |  | A | 0.926 | 0.036 | 0.008 | 5.0E-06 | -0.015 | 0.028 | 0.61 |
|  |  |  | rs17169082 |  | G | 0.920 | -0.032 | 0.007 | 1.0E-05 | 0.043 | 0.022 | 0.05 |
|  |  |  | rs17190618 |  | A | 0.840 | -0.033 | 0.005 | 1.2E-09 | 0.022 | 0.016 | 0.18 |
|  |  |  | rs1807282 |  | A | 0.788 | -0.039 | 0.005 | 8.7E-16 | -0.002 | 0.014 | 0.90 |
|  |  |  |  |  |  |  | Gene-exposure estimate | | | Gene-outcome estimate | | |
| Exposure | Outcome | Threshold genetic instrument | SNP | Original SNP if proxy used (LD R^2^ with original SNP) | Effect allele | Effect allele frequency | beta | SE | p-value | beta | SE | p-value |
|  |  |  | rs242717 |  | T | 0.682 | -0.019 | 0.004 | 8.0E-06 | -0.019 | 0.012 | 0.13 |
|  |  |  | rs2846581 |  | C | 0.275 | -0.024 | 0.004 | 1.2E-07 | 0.002 | 0.013 | 0.87 |
|  |  |  | rs3095508 |  | C | 0.592 | 0.019 | 0.004 | 3.6E-06 | -0.003 | 0.012 | 0.77 |
|  |  |  | rs342745 |  | A | 0.201 | -0.023 | 0.005 | 5.4E-06 | -0.004 | 0.014 | 0.79 |
|  |  |  | rs6425885 |  | G | 0.294 | 0.021 | 0.004 | 1.4E-06 | 0.011 | 0.013 | 0.42 |
|  |  |  | rs6772 |  | C | 0.267 | -0.021 | 0.004 | 3.7E-06 | 0.007 | 0.015 | 0.65 |
|  |  |  | rs6948971 |  | A | 0.804 | -0.025 | 0.005 | 7.2E-07 | 0.030 | 0.015 | 0.05 |
|  |  |  | rs7329346 |  | C | 0.588 | -0.020 | 0.004 | 1.9E-06 | 0.002 | 0.012 | 0.89 |
|  |  |  | rs7827165 |  | C | 0.497 | -0.019 | 0.004 | 3.0E-06 | -0.020 | 0.012 | 0.08 |
|  |  |  | rs7932863 |  | A | 0.269 | 0.021 | 0.005 | 4.4E-06 | -0.008 | 0.017 | 0.62 |
| Sleep duration | Cigarettes per day | *p*<5×10^-8^ | rs1380703 |  | A | 0.618 | 0.025 | 0.004 | 7.6E-09 | -0.058 | 0.089 | 0.52 |
|  |  |  | rs17190618 |  | A | 0.840 | -0.033 | 0.005 | 1.2E-09 | -0.194 | 0.115 | 0.09 |
|  |  |  | rs1807282 | rs62158211 (0.99) | A | 0.788 | -0.039 | 0.005 | 8.7E-16 | -0.042 | 0.099 | 0.67 |
| Sleep duration | Cigarettes per day | *p*<1×10^-5^ | rs10510128 |  | G | 0.795 | -0.022 | 0.005 | 4.6E-06 | 0.158 | 0.099 | 0.11 |
|  |  |  | rs10840160 |  | T | 0.536 | 0.019 | 0.004 | 2.7E-06 | -0.103 | 0.085 | 0.22 |
|  |  |  | rs11152363 |  | G | 0.813 | 0.023 | 0.005 | 8.3E-06 | -0.055 | 0.105 | 0.60 |
|  |  |  | rs11964802 |  | A | 0.712 | 0.019 | 0.004 | 8.5E-06 | 0.164 | 0.160 | 0.31 |
|  |  |  | rs1204056 |  | G | 0.338 | 0.015 | 0.004 | 3.0E-04 | -0.008 | 0.088 | 0.93 |
|  |  |  | rs12984777 |  | C | 0.972 | -0.058 | 0.012 | 2.1E-06 | -0.069 | 0.598 | 0.91 |
|  |  |  | rs1380703 |  | A | 0.618 | 0.025 | 0.004 | 7.6E-09 | -0.058 | 0.089 | 0.52 |
|  |  |  | rs16865859 |  | T | 0.869 | -0.027 | 0.006 | 3.2E-06 | -0.036 | 0.119 | 0.77 |
|  |  |  | rs16949934 |  | G | 0.920 | -0.034 | 0.007 | 3.3E-06 | -0.027 | 0.145 | 0.85 |
|  |  |  | rs17121264 |  | A | 0.926 | 0.036 | 0.008 | 5.0E-06 | 0.120 | 0.203 | 0.55 |
|  |  |  |  |  |  |  | Gene-exposure estimate | | | Gene-outcome estimate | | |
| Exposure | Outcome | Threshold genetic instrument | SNP | Original SNP if proxy used (LD R^2^ with original SNP) | Effect allele | Effect allele frequency | beta | SE | p-value | beta | SE | p-value |
|  |  |  | rs17169082 |  | G | 0.920 | -0.032 | 0.007 | 1.0E-05 | 0.158 | 0.157 | 0.31 |
|  |  |  | rs17190618 |  | A | 0.840 | -0.033 | 0.005 | 1.2E-09 | -0.194 | 0.115 | 0.09 |
|  |  |  | rs1807282 |  | A | 0.788 | -0.039 | 0.005 | 8.7E-16 | -0.042 | 0.099 | 0.67 |
|  |  |  | rs242717 |  | T | 0.682 | -0.019 | 0.004 | 8.0E-06 | -0.005 | 0.087 | 0.96 |
|  |  |  | rs2846581 |  | C | 0.275 | -0.024 | 0.004 | 1.2E-07 | -0.048 | 0.090 | 0.59 |
|  |  |  | rs3095508 |  | C | 0.592 | 0.019 | 0.004 | 3.6E-06 | 0.185 | 0.083 | 0.03 |
|  |  |  | rs342745 |  | A | 0.201 | -0.023 | 0.005 | 5.4E-06 | -0.179 | 0.101 | 0.08 |
|  |  |  | rs6425885 |  | G | 0.294 | 0.021 | 0.004 | 1.4E-06 | -0.111 | 0.091 | 0.22 |
|  |  |  | rs6772 |  | C | 0.267 | -0.021 | 0.004 | 3.7E-06 | -0.073 | 0.102 | 0.47 |
|  |  |  | rs6948971 |  | A | 0.804 | -0.025 | 0.005 | 7.2E-07 | 0.091 | 0.106 | 0.39 |
|  |  |  | rs7329346 |  | C | 0.588 | -0.020 | 0.004 | 1.9E-06 | -0.055 | 0.084 | 0.51 |
|  |  |  | rs7827165 |  | C | 0.497 | -0.019 | 0.004 | 3.0E-06 | -0.021 | 0.082 | 0.80 |
|  |  |  | rs7932863 |  | A | 0.269 | 0.021 | 0.005 | 4.4E-06 | -0.036 | 0.115 | 0.75 |
| Sleep duration | Smoking cessation | *p*<5×10^-8^ | rs1380703 |  | A | 0.618 | 0.025 | 0.004 | 7.6E-09 | 0.012 | 0.018 | 0.49 |
|  |  |  | rs17190618 |  | A | 0.840 | -0.033 | 0.005 | 1.2E-09 | 0.032 | 0.022 | 0.15 |
|  |  |  | rs1807282 | rs62158211 (0.99) | A | 0.788 | -0.039 | 0.005 | 8.7E-16 | 0.010 | 0.020 | 0.62 |
| Sleep duration | Smoking cessation | *p*<1×10^-5^ | rs10510128 |  | G | 0.795 | -0.022 | 0.005 | 4.6E-06 | -0.007 | 0.019 | 0.73 |
|  |  |  | rs10840160 |  | T | 0.536 | 0.019 | 0.004 | 2.7E-06 | 0.009 | 0.016 | 0.57 |
|  |  |  | rs11152363 |  | G | 0.813 | 0.023 | 0.005 | 8.3E-06 | 0.024 | 0.020 | 0.24 |
|  |  |  | rs11964802 |  | A | 0.712 | 0.019 | 0.004 | 8.5E-06 | -0.028 | 0.031 | 0.36 |
|  |  |  | rs1204056 |  | G | 0.338 | 0.015 | 0.004 | 3.0E-04 | -0.034 | 0.017 | 0.04 |
|  |  |  | rs12984777 |  | C | 0.972 | -0.058 | 0.012 | 2.1E-06 | -0.025 | 0.122 | 0.84 |
|  |  |  | rs1380703 |  | A | 0.618 | 0.025 | 0.004 | 7.6E-09 | 0.012 | 0.018 | 0.49 |
|  |  |  |  |  |  |  | Gene-exposure estimate | | | Gene-outcome estimate | | |
| Exposure | Outcome | Threshold genetic instrument | SNP | Original SNP if proxy used (LD R^2^ with original SNP) | Effect allele | Effect allele frequency | beta | SE | p-value | beta | SE | p-value |
|  |  |  | rs16865859 |  | T | 0.869 | -0.027 | 0.006 | 3.2E-06 | -0.022 | 0.023 | 0.35 |
|  |  |  | rs16949934 |  | G | 0.920 | -0.034 | 0.007 | 3.3E-06 | 0.043 | 0.028 | 0.13 |
|  |  |  | rs17121264 |  | A | 0.926 | 0.036 | 0.008 | 5.0E-06 | -0.060 | 0.042 | 0.16 |
|  |  |  | rs17169082 |  | G | 0.920 | -0.032 | 0.007 | 1.0E-05 | -0.010 | 0.031 | 0.75 |
|  |  |  | rs17190618 |  | A | 0.840 | -0.033 | 0.005 | 1.2E-09 | 0.032 | 0.022 | 0.15 |
|  |  |  | rs1807282 |  | A | 0.788 | -0.039 | 0.005 | 8.7E-16 | 0.010 | 0.020 | 0.62 |
|  |  |  | rs242717 |  | T | 0.682 | -0.019 | 0.004 | 8.0E-06 | 0.006 | 0.017 | 0.72 |
|  |  |  | rs2846581 |  | C | 0.275 | -0.024 | 0.004 | 1.2E-07 | 0.022 | 0.018 | 0.21 |
|  |  |  | rs3095508 |  | C | 0.592 | 0.019 | 0.004 | 3.6E-06 | -0.005 | 0.016 | 0.76 |
|  |  |  | rs342745 |  | A | 0.201 | -0.023 | 0.005 | 5.4E-06 | 0.011 | 0.020 | 0.58 |
|  |  |  | rs6425885 |  | G | 0.294 | 0.021 | 0.004 | 1.4E-06 | 0.011 | 0.018 | 0.53 |
|  |  |  | rs6772 |  | C | 0.267 | -0.021 | 0.004 | 3.7E-06 | 0.010 | 0.020 | 0.63 |
|  |  |  | rs6948971 |  | A | 0.804 | -0.025 | 0.005 | 7.2E-07 | -0.004 | 0.021 | 0.86 |
|  |  |  | rs7329346 |  | C | 0.588 | -0.020 | 0.004 | 1.9E-06 | 0.043 | 0.016 | 0.01 |
|  |  |  | rs7827165 |  | C | 0.497 | -0.019 | 0.004 | 3.0E-06 | -0.001 | 0.016 | 0.97 |
|  |  |  | rs7932863 |  | A | 0.269 | 0.021 | 0.005 | 4.4E-06 | 0.004 | 0.023 | 0.88 |
| Undersleeping | Smoking initiation | *p*<1×10^-5^ | rs10139059 | rs8008258 (1.0) | A | 0.569 | -0.008 | 0.002 | 4.3E-06 | 0.001 | 0.012 | 0.97 |
|  |  |  | rs10510128 |  | G | 0.795 | 0.011 | 0.002 | 1.0E-06 | 0.032 | 0.014 | 0.02 |
|  |  |  | rs12195564 | rs12196190 (1.0) | T | 0.884 | 0.013 | 0.003 | 6.7E-06 | -0.041 | 0.019 | 0.03 |
|  |  |  | rs13217514 | rs9474974 (1.0) | T | 0.434 | 0.008 | 0.002 | 1.5E-05 | -0.001 | 0.012 | 0.92 |
|  |  |  | rs1380703 |  | A | 0.617 | -0.010 | 0.002 | 1.2E-06 | 0.026 | 0.013 | 0.04 |
|  |  |  | rs1563408 | rs1456031 (0.82) | G | 0.514 | -0.007 | 0.002 | 3.2E-04 | -0.010 | 0.012 | 0.42 |
|  |  |  | rs16992934 | rs6133379 (0.87) | A | 0.925 | 0.013 | 0.004 | 1.8E-04 | 0.016 | 0.041 | 0.70 |
|  |  |  |  |  |  |  | Gene-exposure estimate | | | Gene-outcome estimate | | |
| Exposure | Outcome | Threshold genetic instrument | SNP | Original SNP if proxy used (LD R^2^ with original SNP) | Effect allele | Effect allele frequency | beta | SE | p-value | beta | SE | p-value |
|  |  |  | rs17135999 | rs75175983 (0.83) | T | 0.908 | -0.011 | 0.003 | 5.3E-04 | -0.003 | 0.021 | 0.90 |
|  |  |  | rs1807282 | rs62158211 (0.99) | A | 0.789 | 0.010 | 0.002 | 4.9E-06 | -0.002 | 0.014 | 0.90 |
|  |  |  | rs2203635 | rs13111539 (0.98) | C | 0.936 | -0.016 | 0.004 | 1.2E-05 | 0.020 | 0.024 | 0.41 |
|  |  |  | rs2826084 | rs1487942 (1.0) | C | 0.140 | -0.012 | 0.003 | 6.6E-06 | -0.010 | 0.016 | 0.55 |
|  |  |  | rs7097157 | rs34219621 (0.95) | T | 0.892 | 0.012 | 0.003 | 2.8E-05 | 0.016 | 0.018 | 0.38 |
|  |  |  | rs7483691 |  | C | 0.286 | -0.010 | 0.002 | 5.1E-07 | 0.009 | 0.013 | 0.51 |
|  |  |  | rs7845535 |  | C | 0.462 | 0.008 | 0.002 | 5.7E-06 | 0.010 | 0.012 | 0.40 |
|  |  |  | rs838528 | rs2094123 (0.90) | G | 0.558 | -0.008 | 0.002 | 1.3E-05 | 0.003 | 0.012 | 0.77 |
| Undersleeping | Cigarettes per day | *p*<1×10^-5^ | rs10139059 | rs8008258 (1.0) | A | 0.569 | -0.008 | 0.002 | 4.3E-06 | 0.047 | 0.082 | 0.57 |
|  |  |  | rs10510128 |  | G | 0.795 | 0.011 | 0.002 | 1.0E-06 | 0.158 | 0.099 | 0.11 |
|  |  |  | rs12195564 | rs12196190 (1.0) | T | 0.884 | 0.013 | 0.003 | 6.7E-06 | 0.102 | 0.132 | 0.44 |
|  |  |  | rs13217514 | rs9474974 (1.0) | T | 0.434 | 0.008 | 0.002 | 1.5E-05 | 0.022 | 0.085 | 0.80 |
|  |  |  | rs1380703 |  | A | 0.617 | -0.010 | 0.002 | 1.2E-06 | -0.058 | 0.089 | 0.52 |
|  |  |  | rs1563408 | rs1456031 (0.82) | G | 0.514 | -0.007 | 0.002 | 3.2E-04 | -0.027 | 0.084 | 0.74 |
|  |  |  | rs16992934 | rs6133379 (0.87) | A | 0.925 | 0.013 | 0.004 | 1.8E-04 | -0.032 | 0.273 | 0.91 |
|  |  |  | rs17135999 | rs75175983 (0.83) | T | 0.908 | -0.011 | 0.003 | 5.3E-04 | -0.004 | 0.146 | 0.98 |
|  |  |  | rs1807282 | rs62158211 (0.99) | A | 0.789 | 0.010 | 0.002 | 4.9E-06 | -0.042 | 0.099 | 0.67 |
|  |  |  | rs2203635 | rs13111539 (0.98) | C | 0.936 | -0.016 | 0.004 | 1.2E-05 | 0.068 | 0.163 | 0.68 |
|  |  |  | rs2826084 | rs1487942 (1.0) | C | 0.140 | -0.012 | 0.003 | 6.6E-06 | -0.179 | 0.114 | 0.11 |
|  |  |  | rs7097157 | rs34219621 (0.95) | T | 0.892 | 0.012 | 0.003 | 2.8E-05 | 0.155 | 0.127 | 0.22 |
|  |  |  | rs7483691 |  | C | 0.286 | -0.010 | 0.002 | 5.1E-07 | 0.089 | 0.092 | 0.33 |
|  |  |  | rs7845535 |  | C | 0.462 | 0.008 | 0.002 | 5.7E-06 | 0.064 | 0.081 | 0.43 |
|  |  |  | rs838528 | rs2094123 (0.90) | G | 0.558 | -0.008 | 0.002 | 1.3E-05 | -0.022 | 0.084 | 0.79 |
|  |  |  |  |  |  |  | Gene-exposure estimate | | | Gene-outcome estimate | | |
| Exposure | Outcome | Threshold genetic instrument | SNP | Original SNP if proxy used (LD R^2^ with original SNP) | Effect allele | Effect allele frequency | beta | SE | p-value | beta | SE | p-value |
| Undersleeping | Smoking cessation | *p*<1×10^-5^ | rs10139059 | rs8008258 (1.0) | A | 0.569 | -0.008 | 0.002 | 4.3E-06 | -0.014 | 0.016 | 0.38 |
|  |  |  | rs10510128 |  | G | 0.795 | 0.011 | 0.002 | 1.0E-06 | -0.007 | 0.019 | 0.73 |
|  |  |  | rs12195564 | rs12196190 (1.0) | T | 0.884 | 0.013 | 0.003 | 6.7E-06 | -0.029 | 0.026 | 0.25 |
|  |  |  | rs13217514 | rs9474974 (1.0) | T | 0.434 | 0.008 | 0.002 | 1.5E-05 | -0.011 | 0.016 | 0.52 |
|  |  |  | rs1380703 |  | A | 0.617 | -0.010 | 0.002 | 1.2E-06 | 0.012 | 0.018 | 0.49 |
|  |  |  | rs1563408 | rs1456031 (0.82) | G | 0.514 | -0.007 | 0.002 | 3.2E-04 | -0.002 | 0.016 | 0.93 |
|  |  |  | rs16992934 | rs6133379 (0.87) | A | 0.925 | 0.013 | 0.004 | 1.8E-04 | 0.007 | 0.052 | 0.89 |
|  |  |  | rs17135999 | rs75175983 (0.83) | T | 0.908 | -0.011 | 0.003 | 5.3E-04 | 0.021 | 0.029 | 0.48 |
|  |  |  | rs1807282 | rs62158211 (0.99) | A | 0.789 | 0.010 | 0.002 | 4.9E-06 | 0.010 | 0.020 | 0.62 |
|  |  |  | rs2203635 | rs13111539 (0.98) | C | 0.936 | -0.016 | 0.004 | 1.2E-05 | -0.035 | 0.032 | 0.27 |
|  |  |  | rs2826084 | rs1487942 (1.0) | C | 0.140 | -0.012 | 0.003 | 6.6E-06 | -0.023 | 0.022 | 0.30 |
|  |  |  | rs7097157 | rs34219621 (0.95) | T | 0.892 | 0.012 | 0.003 | 2.8E-05 | -0.019 | 0.025 | 0.45 |
|  |  |  | rs7483691 |  | C | 0.286 | -0.010 | 0.002 | 5.1E-07 | -0.007 | 0.018 | 0.69 |
|  |  |  | rs7845535 |  | C | 0.462 | 0.008 | 0.002 | 5.7E-06 | -0.011 | 0.016 | 0.50 |
|  |  |  | rs838528 | rs2094123 (0.90) | G | 0.558 | -0.008 | 0.002 | 1.3E-05 | -0.033 | 0.016 | 0.04 |
| Oversleeping | Smoking initiation | *p*<1×10^-5^ | rs10745487 |  | T | 0.571 | 0.006 | 0.001 | 7.4E-06 | -0.006 | 0.012 | 0.62 |
|  |  |  | rs10914398 | rs12751487 (0.85) | T | 0.740 | 0.007 | 0.002 | 3.1E-06 | -0.015 | 0.013 | 0.25 |
|  |  |  | rs13091558 | rs6781801 (1.0) | A | 0.700 | 0.008 | 0.002 | 5.3E-07 | -0.023 | 0.013 | 0.06 |
|  |  |  | rs1471172 |  | G | 0.343 | 0.007 | 0.001 | 1.6E-06 | 0.012 | 0.012 | 0.35 |
|  |  |  | rs2179551 | rs6456355 (0.99) | C | 0.249 | 0.007 | 0.002 | 6.7E-06 | 0.014 | 0.014 | 0.31 |
|  |  |  | rs4399947 |  | A | 0.749 | -0.007 | 0.002 | 8.2E-06 | 0.002 | 0.013 | 0.86 |
|  |  |  | rs6035251 |  | A | 0.071 | 0.013 | 0.003 | 1.1E-06 | -0.031 | 0.030 | 0.29 |
|  |  |  | rs7130539 |  | T | 0.953 | -0.015 | 0.003 | 5.4E-06 | 0.010 | 0.029 | 0.74 |
|  |  |  |  |  |  |  | Gene-exposure estimate | | | Gene-outcome estimate | | |
| Exposure | Outcome | Threshold genetic instrument | SNP | Original SNP if proxy used (LD R^2^ with original SNP) | Effect allele | Effect allele frequency | beta | SE | p-value | beta | SE | p-value |
|  |  |  | rs7792983 | rs41308993 (0.97) | C | 0.976 | 0.018 | 0.005 | 5.6E-05 | 0.023 | 0.042 | 0.58 |
|  |  |  | rs7871719 |  | G | 0.185 | 0.008 | 0.002 | 6.3E-06 | 0.007 | 0.016 | 0.66 |
|  |  |  | rs8065143 | rs9915132 (1.0) | A | 0.017 | -0.025 | 0.005 | 2.9E-06 | -0.034 | 0.040 | 0.39 |
|  |  |  | rs9620654 | rs6005270 (0.87) | G | 0.906 | -0.009 | 0.002 | 8.2E-05 | 0.002 | 0.034 | 0.95 |
| Oversleeping | Cigarettes per day | *p*<1×10^-5^ | rs10745487 |  | T | 0.571 | 0.006 | 0.001 | 7.4E-06 | -0.002 | 0.086 | 0.98 |
|  |  |  | rs10914398 | rs12751487 (0.85) | T | 0.740 | 0.007 | 0.002 | 3.1E-06 | 0.055 | 0.092 | 0.55 |
|  |  |  | rs13091558 | rs6781801 (1.0) | A | 0.700 | 0.008 | 0.002 | 5.3E-07 | -0.043 | 0.088 | 0.62 |
|  |  |  | rs1471172 |  | G | 0.343 | 0.007 | 0.001 | 1.6E-06 | 0.032 | 0.088 | 0.71 |
|  |  |  | rs2179551 | rs6456355 (0.99) | C | 0.249 | 0.007 | 0.002 | 6.7E-06 | -0.017 | 0.096 | 0.86 |
|  |  |  | rs4399947 |  | A | 0.749 | -0.007 | 0.002 | 8.2E-06 | -0.105 | 0.094 | 0.26 |
|  |  |  | rs6035251 |  | A | 0.071 | 0.013 | 0.003 | 1.1E-06 | -0.050 | 0.215 | 0.82 |
|  |  |  | rs7130539 |  | T | 0.953 | -0.015 | 0.003 | 5.4E-06 | 0.106 | 0.203 | 0.60 |
|  |  |  | rs7792983 | rs41308993 (0.97) | C | 0.976 | 0.018 | 0.005 | 5.6E-05 | 0.083 | 0.287 | 0.77 |
|  |  |  | rs7871719 |  | G | 0.185 | 0.008 | 0.002 | 6.3E-06 | 0.191 | 0.114 | 0.10 |
|  |  |  | rs8065143 | rs9915132 (1.0) | A | 0.017 | -0.025 | 0.005 | 2.9E-06 | -0.118 | 0.277 | 0.67 |
|  |  |  | rs9620654 | rs6005270 (0.87) | G | 0.906 | -0.009 | 0.002 | 8.2E-05 | 0.107 | 0.250 | 0.67 |
| Oversleeping | Smoking cessation | *p*<1×10^-5^ | rs10745487 |  | T | 0.571 | 0.006 | 0.001 | 7.4E-06 | -0.014 | 0.017 | 0.39 |
|  |  |  | rs10914398 | rs12751487 (0.85) | T | 0.740 | 0.007 | 0.002 | 3.1E-06 | -0.001 | 0.018 | 0.97 |
|  |  |  | rs13091558 | rs6781801 (1.0) | A | 0.700 | 0.008 | 0.002 | 5.3E-07 | 0.031 | 0.017 | 0.07 |
|  |  |  | rs1471172 |  | G | 0.343 | 0.007 | 0.001 | 1.6E-06 | 0.005 | 0.017 | 0.77 |
|  |  |  | rs2179551 | rs6456355 (0.99) | C | 0.249 | 0.007 | 0.002 | 6.7E-06 | 0.013 | 0.019 | 0.51 |
|  |  |  | rs4399947 |  | A | 0.749 | -0.007 | 0.002 | 8.2E-06 | -0.030 | 0.018 | 0.10 |
|  |  |  | rs6035251 |  | A | 0.071 | 0.013 | 0.003 | 1.1E-06 | -0.141 | 0.040 | 3.90E-04 |
|  |  |  |  |  |  |  | Gene-exposure estimate | | | Gene-outcome estimate | | |
| Exposure | Outcome | Threshold genetic instrument | SNP | Original SNP if proxy used (LD R^2^ with original SNP) | Effect allele | Effect allele frequency | beta | SE | p-value | beta | SE | p-value |
|  |  |  | rs7130539 |  | T | 0.953 | -0.015 | 0.003 | 5.4E-06 | 0.074 | 0.040 | 0.06 |
|  |  |  | rs7792983 | rs41308993 (0.97) | C | 0.976 | 0.018 | 0.005 | 5.6E-05 | -0.030 | 0.060 | 0.61 |
|  |  |  | rs7871719 |  | G | 0.185 | 0.008 | 0.002 | 6.3E-06 | 0.027 | 0.022 | 0.23 |
|  |  |  | rs8065143 | rs9915132 (1.0) | A | 0.017 | -0.025 | 0.005 | 2.9E-06 | 0.067 | 0.053 | 0.21 |
|  |  |  | rs9620654 | rs6005270 (0.87) | G | 0.906 | -0.009 | 0.002 | 8.2E-05 | 0.051 | 0.046 | 0.26 |
| Chronotype | Smoking initiation | *p*<5×10^-8^ | rs10157197 |  | G | 0.602 | 0.025 | 0.004 | 1.0E-09 | 0.002 | 0.012 | 0.85 |
|  |  |  | rs10922910 |  | G | 0.746 | -0.023 | 0.005 | 5.1E-07 | 0.035 | 0.013 | 0.01 |
|  |  |  | rs12040629 | rs113240734 (1.0) | G | 0.839 | -0.037 | 0.005 | 2.4E-12 | 0.015 | 0.015 | 0.34 |
|  |  |  | rs2653349 |  | A | 0.216 | 0.026 | 0.005 | 5.5E-08 | -0.009 | 0.015 | 0.55 |
|  |  |  | rs3769124 |  | G | 0.875 | 0.030 | 0.006 | 2.5E-07 | -0.020 | 0.018 | 0.26 |
|  |  |  | rs4912138 | rs2050122 (1.0) | A | 0.195 | 0.027 | 0.005 | 4.2E-08 | -0.007 | 0.015 | 0.64 |
|  |  |  | rs516134 |  | C | 0.031 | 0.081 | 0.011 | 8.9E-13 | 0.055 | 0.041 | 0.18 |
|  |  |  | rs9961653 |  | T | 0.422 | 0.023 | 0.004 | 9.6E-09 | 0.001 | 0.013 | 0.93 |
| Chronotype | Smoking initiation | *p*<1×10^-5^ | rs10113427 |  | A | 0.763 | 0.022 | 0.005 | 1.7E-06 | -0.013 | 0.014 | 0.33 |
|  |  |  | rs10157197 |  | G | 0.602 | 0.025 | 0.004 | 1.0E-09 | 0.002 | 0.012 | 0.85 |
|  |  |  | rs10269368 |  | A | 0.735 | 0.024 | 0.004 | 9.3E-08 | -0.007 | 0.014 | 0.65 |
|  |  |  | rs10864316 | rs7545893 (0.99) | A | 0.804 | -0.025 | 0.005 | 5.7E-07 | 0.007 | 0.015 | 0.63 |
|  |  |  | rs10931166 | rs62198772 (0.91) | C | 0.604 | 0.016 | 0.004 | 4.4E-05 | 0.003 | 0.012 | 0.80 |
|  |  |  | rs11080887 |  | A | 0.867 | -0.026 | 0.006 | 5.5E-06 | 0.017 | 0.017 | 0.33 |
|  |  |  | rs11596752 | rs78682903 (0.95) | G | 0.889 | -0.027 | 0.006 | 1.2E-05 | 0.027 | 0.026 | 0.31 |
|  |  |  | rs11699264 |  | G | 0.847 | 0.026 | 0.005 | 3.1E-06 | -0.005 | 0.022 | 0.84 |
|  |  |  | rs11841507 | rs75049912 (0.82) | C | 0.964 | -0.043 | 0.011 | 4.3E-05 | -0.009 | 0.033 | 0.80 |
|  |  |  | rs11841797 | rs376908252 (0.96) | A | 0.685 | -0.020 | 0.004 | 1.9E-06 | 0.002 | 0.013 | 0.87 |
|  |  |  |  |  |  |  | Gene-exposure estimate | | | Gene-outcome estimate | | |
| Exposure | Outcome | Threshold genetic instrument | SNP | Original SNP if proxy used (LD R^2^ with original SNP) | Effect allele | Effect allele frequency | beta | SE | p-value | beta | SE | p-value |
|  |  |  | rs12022460 | rs72720396 (0.84) | G | 0.746 | -0.023 | 0.005 | 3.6E-07 | 0.035 | 0.013 | 0.01 |
|  |  |  | rs12040629 | rs113240734 (1.0) | G | 0.839 | -0.037 | 0.005 | 2.4E-12 | 0.015 | 0.015 | 0.34 |
|  |  |  | rs12241819 | rs7903778 (0.93) | G | 0.721 | -0.018 | 0.004 | 2.4E-05 | -0.007 | 0.013 | 0.61 |
|  |  |  | rs12580830 |  | C | 0.481 | 0.019 | 0.004 | 1.7E-06 | -0.011 | 0.012 | 0.36 |
|  |  |  | rs12635403 | rs12635074 (0.97) | C | 0.681 | -0.023 | 0.004 | 7.6E-08 | 0.015 | 0.012 | 0.24 |
|  |  |  | rs12651919 | rs12657877 (0.91) | A | 0.840 | -0.022 | 0.005 | 3.2E-05 | 0.024 | 0.017 | 0.15 |
|  |  |  | rs13133212 |  | G | 0.883 | -0.029 | 0.006 | 1.8E-06 | -0.002 | 0.029 | 0.96 |
|  |  |  | rs13290794 | rs17487601 (0.95) | G | 0.629 | 0.018 | 0.004 | 6.8E-06 | 0.002 | 0.012 | 0.89 |
|  |  |  | rs1347531 | rs8055492 (0.99) | C | 0.633 | -0.019 | 0.004 | 3.4E-06 | 0.006 | 0.012 | 0.61 |
|  |  |  | rs1464776 |  | T | 0.481 | 0.019 | 0.004 | 1.9E-06 | 0.010 | 0.012 | 0.37 |
|  |  |  | rs16939130 |  | T | 0.779 | 0.026 | 0.005 | 6.0E-08 | -0.001 | 0.014 | 0.96 |
|  |  |  | rs17311976 |  | T | 0.810 | 0.025 | 0.005 | 8.6E-07 | -0.016 | 0.017 | 0.34 |
|  |  |  | rs17454584 | rs45515895 (0.97) | A | 0.782 | 0.020 | 0.005 | 2.5E-05 | 0.036 | 0.015 | 0.01 |
|  |  |  | rs17659542 |  | C | 0.846 | -0.028 | 0.005 | 2.7E-07 | -0.009 | 0.017 | 0.59 |
|  |  |  | rs1889060 |  | A | 0.297 | -0.022 | 0.004 | 4.0E-07 | -0.010 | 0.014 | 0.44 |
|  |  |  | rs2035366 | rs1383714 (1.0) | G | 0.796 | -0.021 | 0.005 | 1.3E-05 | 0.018 | 0.014 | 0.20 |
|  |  |  | rs2163761 |  | C | 0.707 | 0.021 | 0.004 | 1.6E-06 | 0.003 | 0.013 | 0.83 |
|  |  |  | rs2244661 | rs2578094 (1.0) | G | 0.265 | 0.019 | 0.004 | 1.2E-05 | 0.005 | 0.013 | 0.69 |
|  |  |  | rs238889 |  | T | 0.591 | -0.019 | 0.004 | 1.3E-06 | 0.020 | 0.012 | 0.10 |
|  |  |  | rs3739070 | rs74409360 (0.93) | A | 0.913 | 0.035 | 0.007 | 3.4E-07 | -0.017 | 0.021 | 0.43 |
|  |  |  | rs3852786 |  | C | 0.505 | 0.019 | 0.004 | 9.1E-07 | 0.000 | 0.012 | 0.97 |
|  |  |  | rs3887436 |  | A | 0.578 | 0.020 | 0.004 | 7.6E-07 | 0.030 | 0.012 | 0.01 |
|  |  |  | rs4245555 | rs4245556 (0.94) | T | 0.588 | -0.020 | 0.004 | 1.2E-06 | 0.005 | 0.012 | 0.69 |
|  |  |  |  |  |  |  | Gene-exposure estimate | | | Gene-outcome estiamte | | |
| Exposure | Outcome | Threshold genetic instrument | SNP | Original SNP if proxy used (LD R^2^ with original SNP) | Effect allele | Effect allele frequency | beta | SE | p-value | beta | SE | p-value |
|  |  |  | rs4662327 |  | G | 0.630 | 0.020 | 0.004 | 6.5E-07 | 0.012 | 0.013 | 0.33 |
|  |  |  | rs4800617 |  | A | 0.401 | -0.019 | 0.004 | 1.8E-06 | -0.017 | 0.012 | 0.16 |
|  |  |  | rs4821940 |  | T | 0.447 | 0.022 | 0.004 | 3.4E-08 | -0.011 | 0.012 | 0.35 |
|  |  |  | rs4912138 | rs2050122 (1.0) | A | 0.195 | 0.027 | 0.005 | 4.2E-08 | -0.007 | 0.015 | 0.64 |
|  |  |  | rs516016 | rs67019387 (1.0) | A | 0.520 | -0.018 | 0.004 | 8.9E-06 | 0.004 | 0.012 | 0.73 |
|  |  |  | rs516134 |  | C | 0.031 | 0.081 | 0.011 | 8.9E-13 | 0.055 | 0.041 | 0.18 |
|  |  |  | rs595877 |  | G | 0.572 | 0.021 | 0.004 | 1.3E-07 | 0.005 | 0.012 | 0.70 |
|  |  |  | rs6002686 |  | G | 0.482 | -0.019 | 0.004 | 1.8E-06 | 0.005 | 0.012 | 0.65 |
|  |  |  | rs6850095 |  | C | 0.904 | 0.032 | 0.007 | 2.0E-06 | 0.019 | 0.020 | 0.35 |
|  |  |  | rs698820 | rs698814 (0.97) | G | 0.236 | -0.022 | 0.005 | 3.1E-06 | 0.006 | 0.014 | 0.69 |
|  |  |  | rs7081035 | rs111623497 (1.0) | C | 0.974 | 0.059 | 0.012 | 1.6E-06 | 0.049 | 0.042 | 0.24 |
|  |  |  | rs7251052 |  | A | 0.818 | -0.023 | 0.005 | 7.1E-06 | -0.007 | 0.015 | 0.65 |
|  |  |  | rs7297861 |  | T | 0.901 | 0.033 | 0.007 | 5.9E-07 | 0.003 | 0.026 | 0.90 |
|  |  |  | rs7342459 |  | T | 0.968 | 0.050 | 0.011 | 5.7E-06 | 0.044 | 0.037 | 0.24 |
|  |  |  | rs7492369 | rs55695162 (0.81) | C | 0.749 | -0.020 | 0.005 | 1.4E-05 | 0.023 | 0.018 | 0.20 |
|  |  |  | rs7563917 |  | C | 0.582 | 0.019 | 0.004 | 1.8E-06 | 0.002 | 0.012 | 0.84 |
|  |  |  | rs7711883 |  | C | 0.844 | -0.024 | 0.005 | 7.5E-06 | -0.013 | 0.016 | 0.43 |
|  |  |  | rs7781395 |  | C | 0.548 | 0.018 | 0.004 | 4.5E-06 | 0.002 | 0.012 | 0.89 |
|  |  |  | rs797148 |  | C | 0.917 | -0.033 | 0.007 | 4.8E-06 | -0.018 | 0.020 | 0.38 |
|  |  |  | rs7976870 |  | G | 0.496 | -0.019 | 0.004 | 1.5E-06 | 0.023 | 0.015 | 0.12 |
|  |  |  | rs872956 |  | T | 0.768 | -0.024 | 0.005 | 4.0E-07 | 0.009 | 0.014 | 0.52 |
|  |  |  | rs9961653 |  | T | 0.422 | 0.023 | 0.004 | 9.6E-09 | 0.001 | 0.013 | 0.93 |
| Chronotype | Cigarettes per day | *p*<5×10^-8^ | rs10157197 |  | G | 0.602 | 0.025 | 0.004 | 1.0E-09 | 0.204 | 0.084 | 0.02 |
|  |  |  |  |  |  |  | Gene-exposure estimate | | | Gene-outcome estimate | | |
| Exposure | Outcome | Threshold genetic instrument | SNP | Original SNP if proxy used (LD R^2^ with original SNP) | Effect allele | Effect allele frequency | beta | SE | p-value | beta | SE | p-value |
|  |  |  | rs10922910 |  | G | 0.746 | -0.023 | 0.005 | 5.1E-07 | 0.208 | 0.094 | 0.03 |
|  |  |  | rs12040629 | rs113240734 (1.0) | G | 0.839 | -0.037 | 0.005 | 2.4E-12 | -0.002 | 0.107 | 0.98 |
|  |  |  | rs2653349 |  | A | 0.216 | 0.026 | 0.005 | 5.5E-08 | 0.149 | 0.102 | 0.14 |
|  |  |  | rs3769124 |  | G | 0.875 | 0.030 | 0.006 | 2.5E-07 | -0.279 | 0.125 | 0.03 |
|  |  |  | rs4912138 | rs2050122 (1.0) | A | 0.195 | 0.027 | 0.005 | 4.2E-08 | -0.015 | 0.103 | 0.88 |
|  |  |  | rs516134 |  | C | 0.031 | 0.081 | 0.011 | 8.9E-13 | -0.211 | 0.287 | 0.46 |
|  |  |  | rs9961653 |  | T | 0.422 | 0.023 | 0.004 | 9.6E-09 | 0.058 | 0.089 | 0.52 |
| Chronotype | Cigarettes per day | *p*<1×10^-5^ | rs10113427 |  | A | 0.763 | 0.022 | 0.005 | 1.7E-06 | 0.192 | 0.096 | 0.05 |
|  |  |  | rs10157197 |  | G | 0.602 | 0.025 | 0.004 | 1.0E-09 | 0.204 | 0.084 | 0.02 |
|  |  |  | rs10269368 |  | A | 0.735 | 0.024 | 0.004 | 9.3E-08 | -0.092 | 0.100 | 0.35 |
|  |  |  | rs10864316 | rs7545893 (0.99) | A | 0.804 | -0.025 | 0.005 | 5.7E-07 | -0.050 | 0.107 | 0.64 |
|  |  |  | rs10931166 | rs62198772 (0.91) | C | 0.604 | 0.016 | 0.004 | 4.4E-05 | -0.017 | 0.083 | 0.84 |
|  |  |  | rs11080887 |  | A | 0.867 | -0.026 | 0.006 | 5.5E-06 | 0.101 | 0.121 | 0.40 |
|  |  |  | rs11596752 | rs78682903 (0.95) | G | 0.889 | -0.027 | 0.006 | 1.2E-05 | 0.033 | 0.180 | 0.86 |
|  |  |  | rs11699264 |  | G | 0.847 | 0.026 | 0.005 | 3.1E-06 | 0.030 | 0.147 | 0.84 |
|  |  |  | rs11841507 | rs75049912 (0.82) | C | 0.964 | -0.043 | 0.011 | 4.3E-05 | -0.035 | 0.226 | 0.88 |
|  |  |  | rs11841797 | rs376908252 (0.96) | A | 0.685 | -0.020 | 0.004 | 1.9E-06 | 0.057 | 0.091 | 0.53 |
|  |  |  | rs12022460 | rs72720396 (0.84) | G | 0.746 | -0.023 | 0.005 | 3.6E-07 | 0.208 | 0.094 | 0.03 |
|  |  |  | rs12040629 | rs113240734 (1.0) | G | 0.839 | -0.037 | 0.005 | 2.4E-12 | -0.002 | 0.107 | 0.98 |
|  |  |  | rs12241819 | rs7903778 (0.93) | G | 0.721 | -0.018 | 0.004 | 2.4E-05 | 0.042 | 0.090 | 0.64 |
|  |  |  | rs12580830 |  | C | 0.481 | 0.019 | 0.004 | 1.7E-06 | -0.130 | 0.083 | 0.12 |
|  |  |  | rs12635403 | rs12635074 (0.97) | C | 0.681 | -0.023 | 0.004 | 7.6E-08 | 0.247 | 0.087 | 0.01 |
|  |  |  | rs12651919 | rs12657877 (0.91) | A | 0.840 | -0.022 | 0.005 | 3.2E-05 | 0.033 | 0.117 | 0.78 |
|  |  |  |  |  |  |  | Gene-exposure estimate | | | Gene-outcome estimate | | |
| Exposure | Outcome | Threshold genetic instrument | SNP | Original SNP if proxy used (LD R^2^ with original SNP) | Effect allele | Effect allele frequency | beta | SE | p-value | beta | SE | p-value |
|  |  |  | rs13133212 |  | G | 0.883 | -0.029 | 0.006 | 1.8E-06 | -0.134 | 0.176 | 0.45 |
|  |  |  | rs13290794 | rs17487601 (0.95) | G | 0.629 | 0.018 | 0.004 | 6.8E-06 | 0.052 | 0.084 | 0.54 |
|  |  |  | rs1347531 | rs8055492 (0.99) | C | 0.633 | -0.019 | 0.004 | 3.4E-06 | 0.043 | 0.084 | 0.61 |
|  |  |  | rs1464776 |  | T | 0.481 | 0.019 | 0.004 | 1.9E-06 | -0.055 | 0.081 | 0.50 |
|  |  |  | rs16939130 |  | T | 0.779 | 0.026 | 0.005 | 6.0E-08 | 0.232 | 0.100 | 0.02 |
|  |  |  | rs17311976 |  | T | 0.810 | 0.025 | 0.005 | 8.6E-07 | 0.115 | 0.113 | 0.31 |
|  |  |  | rs17454584 | rs45515895 (0.97) | A | 0.782 | 0.020 | 0.005 | 2.5E-05 | -0.161 | 0.101 | 0.11 |
|  |  |  | rs17659542 |  | C | 0.846 | -0.028 | 0.005 | 2.7E-07 | 0.149 | 0.119 | 0.21 |
|  |  |  | rs1889060 |  | A | 0.297 | -0.022 | 0.004 | 4.0E-07 | 0.015 | 0.095 | 0.88 |
|  |  |  | rs2035366 | rs1383714 (1.0) | G | 0.796 | -0.021 | 0.005 | 1.3E-05 | -0.014 | 0.097 | 0.89 |
|  |  |  | rs2163761 |  | C | 0.707 | 0.021 | 0.004 | 1.6E-06 | 0.113 | 0.092 | 0.22 |
|  |  |  | rs2244661 | rs2578094 (1.0) | G | 0.265 | 0.019 | 0.004 | 1.2E-05 | 0.095 | 0.094 | 0.31 |
|  |  |  | rs238889 |  | T | 0.591 | -0.019 | 0.004 | 1.3E-06 | -0.047 | 0.085 | 0.58 |
|  |  |  | rs3739070 | rs74409360 (0.93) | A | 0.913 | 0.035 | 0.007 | 3.4E-07 | -0.224 | 0.151 | 0.14 |
|  |  |  | rs3852786 |  | C | 0.505 | 0.019 | 0.004 | 9.1E-07 | 0.132 | 0.081 | 0.10 |
|  |  |  | rs3887436 |  | A | 0.578 | 0.020 | 0.004 | 7.6E-07 | 0.033 | 0.082 | 0.68 |
|  |  |  | rs4245555 | rs4245556 (0.94) | T | 0.588 | -0.020 | 0.004 | 1.2E-06 | 0.147 | 0.084 | 0.08 |
|  |  |  | rs4662327 |  | G | 0.630 | 0.020 | 0.004 | 6.5E-07 | -0.108 | 0.086 | 0.21 |
|  |  |  | rs4800617 |  | A | 0.401 | -0.019 | 0.004 | 1.8E-06 | -0.067 | 0.083 | 0.42 |
|  |  |  | rs4821940 |  | T | 0.447 | 0.022 | 0.004 | 3.4E-08 | 0.014 | 0.083 | 0.86 |
|  |  |  | rs4912138 | rs2050122 (1.0) | A | 0.195 | 0.027 | 0.005 | 4.2E-08 | -0.015 | 0.103 | 0.88 |
|  |  |  | rs516016 | rs67019387 (1.0) | A | 0.520 | -0.018 | 0.004 | 8.9E-06 | -0.048 | 0.082 | 0.55 |
|  |  |  | rs516134 |  | C | 0.031 | 0.081 | 0.011 | 8.9E-13 | -0.211 | 0.287 | 0.46 |
|  |  |  |  |  |  |  | Gene-exposure estimate | | | Gene-outcome estimate | | |
| Exposure | Outcome | Threshold genetic instrument | SNP | Original SNP if proxy used (LD R^2^ with original SNP) | Effect allele | Effect allele frequency | beta | SE | p-value | beta | SE | p-value |
|  |  |  | rs595877 |  | G | 0.572 | 0.021 | 0.004 | 1.3E-07 | 0.102 | 0.086 | 0.24 |
|  |  |  | rs6002686 |  | G | 0.482 | -0.019 | 0.004 | 1.8E-06 | 0.127 | 0.083 | 0.13 |
|  |  |  | rs6850095 |  | C | 0.904 | 0.032 | 0.007 | 2.0E-06 | -0.218 | 0.143 | 0.13 |
|  |  |  | rs698820 | rs698814 (0.97) | G | 0.236 | -0.022 | 0.005 | 3.1E-06 | -0.090 | 0.101 | 0.37 |
|  |  |  | rs7081035 | rs111623497 (1.0) | C | 0.974 | 0.059 | 0.012 | 1.6E-06 | 0.584 | 0.285 | 0.04 |
|  |  |  | rs7251052 |  | A | 0.818 | -0.023 | 0.005 | 7.1E-06 | 0.019 | 0.104 | 0.86 |
|  |  |  | rs7297861 |  | T | 0.901 | 0.033 | 0.007 | 5.9E-07 | 0.189 | 0.169 | 0.27 |
|  |  |  | rs7342459 |  | T | 0.968 | 0.050 | 0.011 | 5.7E-06 | -0.087 | 0.255 | 0.73 |
|  |  |  | rs7492369 | rs55695162 (0.81) | C | 0.749 | -0.020 | 0.005 | 1.4E-05 | -0.120 | 0.128 | 0.35 |
|  |  |  | rs7563917 |  | C | 0.582 | 0.019 | 0.004 | 1.8E-06 | 0.095 | 0.082 | 0.25 |
|  |  |  | rs7711883 |  | C | 0.844 | -0.024 | 0.005 | 7.5E-06 | -0.128 | 0.114 | 0.27 |
|  |  |  | rs7781395 |  | C | 0.548 | 0.018 | 0.004 | 4.5E-06 | -0.025 | 0.082 | 0.76 |
|  |  |  | rs797148 |  | C | 0.917 | -0.033 | 0.007 | 4.8E-06 | 0.017 | 0.142 | 0.91 |
|  |  |  | rs7976870 |  | G | 0.496 | -0.019 | 0.004 | 1.5E-06 | 0.020 | 0.098 | 0.84 |
|  |  |  | rs872956 |  | T | 0.768 | -0.024 | 0.005 | 4.0E-07 | -0.275 | 0.097 | 0.01 |
|  |  |  | rs9961653 |  | T | 0.422 | 0.023 | 0.004 | 9.6E-09 | 0.058 | 0.089 | 0.52 |
| Chronotype | Smoking cessation | *p*<5×10^-8^ | rs10157197 |  | G | 0.602 | 0.025 | 0.004 | 1.0E-09 | 0.005 | 0.016 | 0.77 |
|  |  |  | rs10922910 |  | G | 0.746 | -0.023 | 0.005 | 5.1E-07 | 0.007 | 0.018 | 0.71 |
|  |  |  | rs12040629 | rs113240734 (1.0) | G | 0.839 | -0.037 | 0.005 | 2.4E-12 | 0.023 | 0.021 | 0.28 |
|  |  |  | rs2653349 |  | A | 0.216 | 0.026 | 0.005 | 5.5E-08 | 0.002 | 0.020 | 0.94 |
|  |  |  | rs3769124 |  | G | 0.875 | 0.030 | 0.006 | 2.5E-07 | 0.007 | 0.024 | 0.78 |
|  |  |  | rs4912138 | rs2050122 (1.0) | A | 0.195 | 0.027 | 0.005 | 4.2E-08 | 0.015 | 0.020 | 0.46 |
|  |  |  | rs516134 |  | C | 0.031 | 0.081 | 0.011 | 8.9E-13 | -0.026 | 0.056 | 0.64 |
|  |  |  |  |  |  |  | Gene-exposure estimate | | | Gene-outcome estimate | | |
| Exposure | Outcome | Threshold genetic instrument | SNP | Original SNP if proxy used (LD R^2^ with original SNP) | Effect allele | Effect allele frequency | beta | SE | p-value | beta | SE | p-value |
|  |  |  | rs9961653 |  | T | 0.422 | 0.023 | 0.004 | 9.6E-09 | 0.017 | 0.018 | 0.35 |
| Chronotype | Smoking cessation | *p*<1×10^-5^ | rs10113427 |  | A | 0.763 | 0.022 | 0.005 | 1.7E-06 | -0.033 | 0.019 | 0.07 |
|  |  |  | rs10157197 |  | G | 0.602 | 0.025 | 0.004 | 1.0E-09 | 0.005 | 0.016 | 0.77 |
|  |  |  | rs10269368 |  | A | 0.735 | 0.024 | 0.004 | 9.3E-08 | -0.006 | 0.019 | 0.75 |
|  |  |  | rs10864316 | rs7545893 (0.99) | A | 0.804 | -0.025 | 0.005 | 5.7E-07 | 0.001 | 0.021 | 0.97 |
|  |  |  | rs10931166 | rs62198772 (0.91) | C | 0.604 | 0.016 | 0.004 | 4.4E-05 | -0.015 | 0.016 | 0.38 |
|  |  |  | rs11080887 |  | A | 0.867 | -0.026 | 0.006 | 5.5E-06 | -0.022 | 0.024 | 0.36 |
|  |  |  | rs11596752 | rs78682903 (0.95) | G | 0.889 | -0.027 | 0.006 | 1.2E-05 | 0.004 | 0.033 | 0.91 |
|  |  |  | rs11699264 |  | G | 0.847 | 0.026 | 0.005 | 3.1E-06 | -0.033 | 0.028 | 0.24 |
|  |  |  | rs11841507 | rs75049912 (0.82) | C | 0.964 | -0.043 | 0.011 | 4.3E-05 | -0.011 | 0.044 | 0.80 |
|  |  |  | rs11841797 | rs376908252 (0.96) | A | 0.685 | -0.020 | 0.004 | 1.9E-06 | -0.005 | 0.018 | 0.77 |
|  |  |  | rs12022460 | rs72720396 (0.84) | G | 0.746 | -0.023 | 0.005 | 3.6E-07 | 0.007 | 0.018 | 0.72 |
|  |  |  | rs12040629 | rs113240734 (1.0) | G | 0.839 | -0.037 | 0.005 | 2.4E-12 | 0.023 | 0.021 | 0.28 |
|  |  |  | rs12241819 | rs7903778 (0.93) | G | 0.721 | -0.018 | 0.004 | 2.4E-05 | -0.011 | 0.018 | 0.52 |
|  |  |  | rs12580830 |  | C | 0.481 | 0.019 | 0.004 | 1.7E-06 | -0.002 | 0.016 | 0.92 |
|  |  |  | rs12635403 | rs12635074 (0.97) | C | 0.681 | -0.023 | 0.004 | 7.6E-08 | -0.005 | 0.017 | 0.76 |
|  |  |  | rs12651919 | rs12657877 (0.91) | A | 0.840 | -0.022 | 0.005 | 3.2E-05 | 0.001 | 0.023 | 0.98 |
|  |  |  | rs13133212 |  | G | 0.883 | -0.029 | 0.006 | 1.8E-06 | -0.042 | 0.036 | 0.24 |
|  |  |  | rs13290794 | rs17487601 (0.95) | G | 0.629 | 0.018 | 0.004 | 6.8E-06 | 0.007 | 0.016 | 0.69 |
|  |  |  | rs1347531 | rs8055492 (0.99) | C | 0.633 | -0.019 | 0.004 | 3.4E-06 | -0.026 | 0.017 | 0.11 |
|  |  |  | rs1464776 |  | T | 0.481 | 0.019 | 0.004 | 1.9E-06 | -0.005 | 0.016 | 0.77 |
|  |  |  | rs16939130 |  | T | 0.779 | 0.026 | 0.005 | 6.0E-08 | -0.008 | 0.019 | 0.67 |
|  |  |  | rs17311976 |  | T | 0.810 | 0.025 | 0.005 | 8.6E-07 | 0.011 | 0.022 | 0.63 |
|  |  |  |  |  |  |  | Gene-exposure estimate | | | Gene-outcome estimate | | |
| Exposure | Outcome | Threshold genetic instrument | SNP | Original SNP if proxy used (LD R^2^ with original SNP) | Effect allele | Effect allele frequency | beta | SE | p-value | beta | SE | p-value |
|  |  |  | rs17454584 | rs45515895 (0.97) | A | 0.782 | 0.020 | 0.005 | 2.5E-05 | -0.034 | 0.020 | 0.09 |
|  |  |  | rs17659542 |  | C | 0.846 | -0.028 | 0.005 | 2.7E-07 | 0.038 | 0.023 | 0.10 |
|  |  |  | rs1889060 |  | A | 0.297 | -0.022 | 0.004 | 4.0E-07 | 0.030 | 0.018 | 0.10 |
|  |  |  | rs2035366 | rs1383714 (1.0) | G | 0.796 | -0.021 | 0.005 | 1.3E-05 | -0.007 | 0.019 | 0.70 |
|  |  |  | rs2163761 |  | C | 0.707 | 0.021 | 0.004 | 1.6E-06 | -0.010 | 0.018 | 0.60 |
|  |  |  | rs2244661 | rs2578094 (1.0) | G | 0.265 | 0.019 | 0.004 | 1.2E-05 | -0.003 | 0.018 | 0.86 |
|  |  |  | rs238889 |  | T | 0.591 | -0.019 | 0.004 | 1.3E-06 | -0.001 | 0.016 | 0.93 |
|  |  |  | rs3739070 | rs74409360 (0.93) | A | 0.913 | 0.035 | 0.007 | 3.4E-07 | -0.012 | 0.030 | 0.68 |
|  |  |  | rs3852786 |  | C | 0.505 | 0.019 | 0.004 | 9.1E-07 | -0.011 | 0.016 | 0.50 |
|  |  |  | rs3887436 |  | A | 0.578 | 0.020 | 0.004 | 7.6E-07 | -0.036 | 0.016 | 0.03 |
|  |  |  | rs4245555 | rs4245556 (0.94) | T | 0.588 | -0.020 | 0.004 | 1.2E-06 | 0.021 | 0.016 | 0.20 |
|  |  |  | rs4662327 |  | G | 0.630 | 0.020 | 0.004 | 6.5E-07 | 0.032 | 0.017 | 0.06 |
|  |  |  | rs4800617 |  | A | 0.401 | -0.019 | 0.004 | 1.8E-06 | -0.037 | 0.016 | 0.02 |
|  |  |  | rs4821940 |  | T | 0.447 | 0.022 | 0.004 | 3.4E-08 | 0.007 | 0.016 | 0.66 |
|  |  |  | rs4912138 | rs2050122 (1.0) | A | 0.195 | 0.027 | 0.005 | 4.2E-08 | 0.015 | 0.020 | 0.46 |
|  |  |  | rs516016 | rs67019387 (1.0) | A | 0.520 | -0.018 | 0.004 | 8.9E-06 | 0.007 | 0.016 | 0.65 |
|  |  |  | rs516134 |  | C | 0.031 | 0.081 | 0.011 | 8.9E-13 | -0.026 | 0.056 | 0.64 |
|  |  |  | rs595877 |  | G | 0.572 | 0.021 | 0.004 | 1.3E-07 | -0.025 | 0.017 | 0.14 |
|  |  |  | rs6002686 |  | G | 0.482 | -0.019 | 0.004 | 1.8E-06 | 0.033 | 0.016 | 0.04 |
|  |  |  | rs6850095 |  | C | 0.904 | 0.032 | 0.007 | 2.0E-06 | 0.027 | 0.028 | 0.33 |
|  |  |  | rs698820 | rs698814 (0.97) | G | 0.236 | -0.022 | 0.005 | 3.1E-06 | -0.005 | 0.019 | 0.80 |
|  |  |  | rs7081035 | rs111623497 (1.0) | C | 0.974 | 0.059 | 0.012 | 1.6E-06 | -0.009 | 0.056 | 0.88 |
|  |  |  | rs7251052 |  | A | 0.818 | -0.023 | 0.005 | 7.1E-06 | -0.013 | 0.020 | 0.51 |
|  |  |  |  |  |  |  | Gene-exposure estimate | | | Gene-outcome estimate | | |
| Exposure | Outcome | Threshold genetic instrument | SNP | Original SNP if proxy used (LD R^2^ with original SNP) | Effect allele | Effect allele frequency | beta | SE | p-value | beta | SE | p-value |
|  |  |  | rs7297861 |  | T | 0.901 | 0.033 | 0.007 | 5.9E-07 | -0.016 | 0.033 | 0.62 |
|  |  |  | rs7342459 |  | T | 0.968 | 0.050 | 0.011 | 5.7E-06 | 0.087 | 0.050 | 0.08 |
|  |  |  | rs7492369 | rs55695162 (0.81) | C | 0.749 | -0.020 | 0.005 | 1.4E-05 | 0.025 | 0.026 | 0.34 |
|  |  |  | rs7563917 |  | C | 0.582 | 0.019 | 0.004 | 1.8E-06 | -0.020 | 0.016 | 0.23 |
|  |  |  | rs7711883 |  | C | 0.844 | -0.024 | 0.005 | 7.5E-06 | 0.028 | 0.022 | 0.21 |
|  |  |  | rs7781395 |  | C | 0.548 | 0.018 | 0.004 | 4.5E-06 | 0.006 | 0.016 | 0.73 |
|  |  |  | rs797148 |  | C | 0.917 | -0.033 | 0.007 | 4.8E-06 | -0.005 | 0.028 | 0.85 |
|  |  |  | rs7976870 |  | G | 0.496 | -0.019 | 0.004 | 1.5E-06 | -0.002 | 0.018 | 0.92 |
|  |  |  | rs872956 |  | T | 0.768 | -0.024 | 0.005 | 4.0E-07 | 0.046 | 0.019 | 0.02 |
|  |  |  | rs9961653 |  | T | 0.422 | 0.023 | 0.004 | 9.6E-09 | 0.017 | 0.018 | 0.35 |
| Insomnia | Smoking initiation | *p*<5×10^-8^ | rs11693221 | rs115087496 (0.96) | T | 0.048 | 0.171 | 0.023 | 3.8E-14 | -0.007 | 0.048 | 0.89 |
| Insomnia | Smoking initiation | *p*<1×10^-8^ | rs10250103 |  | T | 0.479 | 0.047 | 0.010 | 6.3E-07 | 0.010 | 0.012 | 0.43 |
|  |  |  | rs11039701 | rs1994140 (1.0) | G | 0.564 | -0.045 | 0.009 | 1.6E-06 | -0.001 | 0.012 | 0.92 |
|  |  |  | rs11653468 | rs72835405 (1.0) | A | 0.233 | -0.049 | 0.011 | 9.1E-06 | 0.008 | 0.024 | 0.76 |
|  |  |  | rs1547668 |  | G | 0.796 | -0.054 | 0.012 | 3.6E-06 | 0.012 | 0.015 | 0.44 |
|  |  |  | rs16966956 | rs4772694 (1.0) | G | 0.281 | -0.047 | 0.010 | 8.7E-06 | -0.019 | 0.015 | 0.21 |
|  |  |  | rs17191759 | rs145265872 (0.80) | C | 0.042 | -0.107 | 0.023 | 4.4E-06 | -0.004 | 0.037 | 0.92 |
|  |  |  | rs17400178 |  | T | 0.071 | 0.084 | 0.019 | 6.6E-06 | 0.019 | 0.026 | 0.48 |
|  |  |  | rs17482814 | rs72664496 (0.88) | C | 0.070 | -0.061 | 0.018 | 8.2E-04 | -0.032 | 0.041 | 0.44 |
|  |  |  | rs2087000 |  | C | 0.223 | 0.051 | 0.011 | 8.0E-06 | -0.025 | 0.014 | 0.08 |
|  |  |  | rs208827 |  | G | 0.636 | 0.051 | 0.010 | 2.2E-07 | -0.031 | 0.013 | 0.02 |
|  |  |  | rs2219778 | rs149326099 (0.88) | T | 0.039 | -0.105 | 0.024 | 1.5E-05 | -0.004 | 0.030 | 0.90 |
|  |  |  | rs2673609 |  | G | 0.360 | 0.045 | 0.010 | 5.0E-06 | -0.022 | 0.012 | 0.07 |
|  |  |  |  |  |  |  | Gene-exposure estimate | | | Gene-outcome estimate | | |
| Exposure | Outcome | Threshold genetic instrument | SNP | Original SNP if proxy used (LD R^2^ with original SNP) | Effect allele | Effect allele frequency | beta | SE | p-value | beta | SE | p-value |
|  |  |  | rs5752673 | rs738475 (0.83) | T | 0.242 | 0.049 | 0.011 | 7.8E-06 | 0.005 | 0.014 | 0.72 |
|  |  |  | rs641783 | rs865199 (0.96) | A | 0.065 | -0.086 | 0.019 | 7.4E-06 | 0.021 | 0.024 | 0.39 |
|  |  |  | rs7332332 |  | G | 0.497 | -0.044 | 0.009 | 3.5E-06 | 0.016 | 0.012 | 0.19 |
|  |  |  | rs7423933 |  | A | 0.735 | -0.047 | 0.011 | 9.1E-06 | -0.027 | 0.014 | 0.05 |
| Insomnia | Cigarettes per day | *p*<5×10^-8^ | rs11693221 | rs115087496 (0.96) | T | 0.048 | 0.171 | 0.023 | 3.8E-14 | -0.266 | 0.368 | 0.47 |
| Insomnia | Cigarettes per day | *p*<1×10^-5^ | rs10250103 |  | T | 0.479 | 0.047 | 0.010 | 6.3E-07 | 0.029 | 0.085 | 0.74 |
|  |  |  | rs11039701 | rs1994140 (1.0) | G | 0.564 | -0.045 | 0.009 | 1.6E-06 | -0.065 | 0.083 | 0.43 |
|  |  |  | rs11653468 | rs72835405 (1.0) | A | 0.233 | -0.049 | 0.011 | 9.1E-06 | -0.067 | 0.170 | 0.70 |
|  |  |  | rs1547668 |  | G | 0.796 | -0.054 | 0.012 | 3.6E-06 | -0.211 | 0.106 | 0.05 |
|  |  |  | rs16966956 | rs4772694 (1.0) | G | 0.281 | -0.047 | 0.010 | 8.7E-06 | -0.044 | 0.100 | 0.66 |
|  |  |  | rs17191759 | rs145265872 (0.80) | C | 0.042 | -0.107 | 0.023 | 4.4E-06 | 0.147 | 0.251 | 0.56 |
|  |  |  | rs17400178 |  | T | 0.071 | 0.084 | 0.019 | 6.6E-06 | -0.024 | 0.186 | 0.90 |
|  |  |  | rs17482814 | rs72664496 (0.88) | C | 0.070 | -0.061 | 0.018 | 8.2E-04 | 0.119 | 0.276 | 0.67 |
|  |  |  | rs2087000 |  | C | 0.223 | 0.051 | 0.011 | 8.0E-06 | 0.086 | 0.101 | 0.40 |
|  |  |  | rs208827 |  | G | 0.636 | 0.051 | 0.010 | 2.2E-07 | 0.215 | 0.088 | 0.02 |
|  |  |  | rs2219778 | rs149326099 (0.88) | T | 0.039 | -0.105 | 0.024 | 1.5E-05 | -0.181 | 0.212 | 0.39 |
|  |  |  | rs2673609 |  | G | 0.360 | 0.045 | 0.010 | 5.0E-06 | -0.009 | 0.086 | 0.91 |
|  |  |  | rs5752673 | rs738475 (0.83) | T | 0.242 | 0.049 | 0.011 | 7.8E-06 | 0.068 | 0.095 | 0.47 |
|  |  |  | rs641783 | rs865199 (0.96) | A | 0.065 | -0.086 | 0.019 | 7.4E-06 | 0.062 | 0.172 | 0.72 |
|  |  |  | rs7332332 |  | G | 0.497 | -0.044 | 0.009 | 3.5E-06 | -0.152 | 0.084 | 0.07 |
|  |  |  | rs7423933 |  | A | 0.735 | -0.047 | 0.011 | 9.1E-06 | 0.060 | 0.094 | 0.53 |
| Insomnia | Cigarettes per day – 2^nd^ release UK biobank* | *p*<1×10^-5^ | rs10250103 |  | T | 0.479 | 0.047 | 0.010 | 6.3E-07 | 0.054 | 0.052 | 0.30 |
|  |  |  | rs10927316 |  | T | 0.309 | -0.046 | 0.010 | 6.8E-06 | -0.028 | 0.056 | 0.62 |
|  |  |  |  |  |  |  | Gene-exposure estimate | | | Gene-outcome estimate | | |
| Exposure | Outcome | Threshold genetic instrument | SNP | Original SNP if proxy used (LD R^2^ with original SNP) | Effect allele | Effect allele frequency | beta | SE | p-value | beta | SE | p-value |
|  |  |  | rs112383419 |  | A | 0.061 | 0.094 | 0.020 | 2.4E-06 | 0.111 | 0.109 | 0.31 |
|  |  |  | rs112974218 |  | T | 0.034 | -0.125 | 0.028 | 9.9E-06 | 0.169 | 0.152 | 0.27 |
|  |  |  | rs113603788 |  | T | 0.011 | -0.203 | 0.046 | 8.6E-06 | -0.103 | 0.246 | 0.68 |
|  |  |  | rs113851554 |  | T | 0.056 | 0.178 | 0.020 | 2.1E-18 | -0.146 | 0.116 | 0.21 |
|  |  |  | rs114740697 |  | G | 0.026 | 0.143 | 0.031 | 4.6E-06 | 0.236 | 0.174 | 0.17 |
|  |  |  | rs115780514 |  | G | 0.019 | -0.166 | 0.034 | 1.2E-06 | -0.033 | 0.189 | 0.86 |
|  |  |  | rs116523352 |  | T | 0.019 | -0.174 | 0.036 | 1.8E-06 | 0.068 | 0.204 | 0.74 |
|  |  |  | rs117149835 |  | C | 0.013 | 0.198 | 0.043 | 3.5E-06 | 0.353 | 0.241 | 0.14 |
|  |  |  | rs117998078 |  | A | 0.016 | 0.169 | 0.038 | 7.0E-06 | -0.143 | 0.218 | 0.51 |
|  |  |  | rs140007258 |  | A | 0.017 | 0.177 | 0.038 | 2.9E-06 | 0.185 | 0.212 | 0.38 |
|  |  |  | rs144669103 |  | T | 0.046 | -0.103 | 0.023 | 5.0E-06 | -0.061 | 0.124 | 0.62 |
|  |  |  | rs145265872 |  | T | 0.046 | -0.107 | 0.023 | 3.0E-06 | 0.048 | 0.125 | 0.70 |
|  |  |  | rs147256205 |  | A | 0.014 | 0.188 | 0.041 | 4.6E-06 | -0.105 | 0.225 | 0.64 |
|  |  |  | rs149251474 |  | G | 0.022 | 0.158 | 0.034 | 2.8E-06 | -0.047 | 0.194 | 0.81 |
|  |  |  | rs149326099 |  | C | 0.036 | -0.115 | 0.026 | 8.6E-06 | -0.182 | 0.137 | 0.18 |
|  |  |  | rs1547668 |  | G | 0.796 | -0.054 | 0.012 | 3.6E-06 | -0.032 | 0.064 | 0.62 |
|  |  |  | rs1552291 |  | G | 0.629 | 0.047 | 0.010 | 1.9E-06 | 0.053 | 0.054 | 0.33 |
|  |  |  | rs17400178 |  | T | 0.071 | 0.084 | 0.019 | 6.6E-06 | -0.022 | 0.103 | 0.83 |
|  |  |  | rs1994140 |  | G | 0.565 | -0.046 | 0.009 | 1.0E-06 | -0.022 | 0.052 | 0.67 |
|  |  |  | rs2087000 |  | C | 0.223 | 0.051 | 0.011 | 8.0E-06 | -0.046 | 0.062 | 0.47 |
|  |  |  | rs208827 |  | G | 0.636 | 0.051 | 0.010 | 2.2E-07 | 0.168 | 0.054 | 0.002 |
|  |  |  | rs2206301 |  | C | 0.625 | -0.050 | 0.010 | 3.2E-07 | 0.059 | 0.054 | 0.27 |
|  |  |  | rs2673609 |  | G | 0.360 | 0.045 | 0.010 | 5.0E-06 | -0.004 | 0.054 | 0.94 |
|  |  |  |  |  |  |  | Gene-exposure estimate | | | Gene-outcome estimate | | |
| Exposure | Outcome | Threshold genetic instrument | SNP | Original SNP if proxy used (LD R^2^ with original SNP) | Effect allele | Effect allele frequency | beta | SE | p-value | beta | SE | p-value |
|  |  |  | rs4772694 |  | T | 0.280 | -0.046 | 0.010 | 8.6E-06 | 0.066 | 0.058 | 0.25 |
|  |  |  | rs618932 |  | G | 0.994 | 0.264 | 0.060 | 9.2E-06 | 0.123 | 0.310 | 0.69 |
|  |  |  | rs62515690 |  | A | 0.015 | 0.199 | 0.043 | 4.6E-06 | -0.129 | 0.214 | 0.55 |
|  |  |  | rs62525086 |  | C | 0.056 | 0.093 | 0.020 | 6.0E-06 | -0.058 | 0.112 | 0.60 |
|  |  |  | rs6437732 |  | T | 0.511 | -0.042 | 0.009 | 6.6E-06 | -0.085 | 0.052 | 0.10 |
|  |  |  | rs72664496 |  | A | 0.066 | -0.085 | 0.019 | 9.3E-06 | 0.100 | 0.104 | 0.34 |
|  |  |  | rs72802225 |  | G | 0.014 | 0.204 | 0.043 | 1.9E-06 | -0.104 | 0.240 | 0.67 |
|  |  |  | rs72835405 |  | T | 0.233 | -0.051 | 0.011 | 6.2E-06 | -0.015 | 0.061 | 0.81 |
|  |  |  | rs7332332 |  | G | 0.497 | -0.044 | 0.009 | 3.5E-06 | 0.038 | 0.052 | 0.47 |
|  |  |  | rs738475 |  | G | 0.259 | 0.051 | 0.011 | 2.5E-06 | 0.026 | 0.059 | 0.66 |
|  |  |  | rs7423933 |  | A | 0.735 | -0.047 | 0.011 | 9.1E-06 | 0.118 | 0.059 | 0.04 |
|  |  |  | rs79278977 |  | T | 0.014 | -0.194 | 0.042 | 3.5E-06 | 0.282 | 0.230 | 0.22 |
|  |  |  | rs865199 |  | G | 0.932 | 0.086 | 0.019 | 4.1E-06 | -0.004 | 0.102 | 0.97 |
| Insomnia | Smoking cessation | *p*<5×10^-8^ | rs11693221 | rs115087496 (0.96) | T | 0.048 | 0.171 | 0.023 | 3.8E-14 | -0.034 | 0.076 | 0.66 |
| Insomnia | Smoking cessation | *p*<1×10^-5^ | rs10250103 |  | T | 0.479 | 0.047 | 0.010 | 6.3E-07 | 0.002 | 0.017 | 0.90 |
|  |  |  | rs11039701 | rs1994140 (1.0) | G | 0.564 | -0.045 | 0.009 | 1.6E-06 | 0.024 | 0.016 | 0.14 |
|  |  |  | rs11653468 | rs72835405 (1.0) | A | 0.233 | -0.049 | 0.011 | 9.1E-06 | 0.020 | 0.031 | 0.51 |
|  |  |  | rs1547668 |  | G | 0.796 | -0.054 | 0.012 | 3.6E-06 | 0.065 | 0.020 | 0.001 |
|  |  |  | rs16966956 | rs4772694 (1.0) | G | 0.281 | -0.047 | 0.010 | 8.7E-06 | 0.010 | 0.021 | 0.62 |
|  |  |  | rs17191759 | rs145265872 (0.80) | C | 0.042 | -0.107 | 0.023 | 4.4E-06 | 0.038 | 0.047 | 0.42 |
|  |  |  | rs17400178 |  | T | 0.071 | 0.084 | 0.019 | 6.6E-06 | 0.002 | 0.036 | 0.97 |
|  |  |  | rs17482814 | rs72664496 (0.88) | C | 0.070 | -0.061 | 0.018 | 8.2E-04 | 0.063 | 0.057 | 0.27 |
|  |  |  | rs2087000 |  | C | 0.223 | 0.051 | 0.011 | 8.0E-06 | 0.015 | 0.020 | 0.45 |
|  |  |  |  |  |  |  | Gene-exposure estimate | | | Gene-outcome estimate | | |
| Exposure | Outcome | Threshold genetic instrument | SNP | Original SNP if proxy used (LD R^2^ with original SNP) | Effect allele | Effect allele frequency | beta | SE | p-value | beta | SE | p-value |
|  |  |  | rs208827 |  | G | 0.636 | 0.051 | 0.010 | 2.2E-07 | -0.002 | 0.017 | 0.92 |
|  |  |  | rs2219778 | rs149326099 (0.88) | T | 0.039 | -0.105 | 0.024 | 1.5E-05 | 0.055 | 0.041 | 0.18 |
|  |  |  | rs2673609 |  | G | 0.360 | 0.045 | 0.010 | 5.0E-06 | 0.015 | 0.017 | 0.38 |
|  |  |  | rs5752673 | rs738475 (0.83) | T | 0.242 | 0.049 | 0.011 | 7.8E-06 | -0.008 | 0.018 | 0.65 |
|  |  |  | rs641783 | rs865199 (0.96) | A | 0.065 | -0.086 | 0.019 | 7.4E-06 | 0.046 | 0.033 | 0.17 |
|  |  |  | rs7332332 |  | G | 0.497 | -0.044 | 0.009 | 3.5E-06 | 0.006 | 0.017 | 0.70 |
|  |  |  | rs7423933 |  | A | 0.735 | -0.047 | 0.011 | 9.1E-06 | 0.003 | 0.018 | 0.89 |
| Insomnia | Smoking cessation – 2^nd^ release UK biobank* | *p*<1×10^-5^ | rs10250103 |  | T | 0.479 | 0.047 | 0.010 | 6.3E-07 | -0.005 | 0.011 | 0.65 |
|  |  |  | rs10927316 |  | T | 0.309 | -0.046 | 0.010 | 6.8E-06 | 0.003 | 0.012 | 0.81 |
|  |  |  | rs112383419 |  | A | 0.061 | 0.094 | 0.020 | 2.4E-06 | -0.029 | 0.023 | 0.20 |
|  |  |  | rs112974218 |  | T | 0.034 | -0.125 | 0.028 | 9.9E-06 | -0.035 | 0.032 | 0.27 |
|  |  |  | rs113603788 |  | T | 0.011 | -0.203 | 0.046 | 8.6E-06 | 0.096 | 0.053 | 0.07 |
|  |  |  | rs113851554 |  | T | 0.056 | 0.178 | 0.020 | 2.1E-18 | -0.026 | 0.024 | 0.29 |
|  |  |  | rs114740697 |  | G | 0.026 | 0.143 | 0.031 | 4.6E-06 | -0.006 | 0.036 | 0.87 |
|  |  |  | rs115780514 |  | G | 0.019 | -0.166 | 0.034 | 1.2E-06 | -0.004 | 0.040 | 0.92 |
|  |  |  | rs116523352 |  | T | 0.019 | -0.174 | 0.036 | 1.8E-06 | -0.012 | 0.042 | 0.78 |
|  |  |  | rs117149835 |  | C | 0.013 | 0.198 | 0.043 | 3.5E-06 | 0.009 | 0.051 | 0.86 |
|  |  |  | rs117998078 |  | A | 0.016 | 0.169 | 0.038 | 7.0E-06 | 0.000 | 0.045 | 0.99 |
|  |  |  | rs140007258 |  | A | 0.017 | 0.177 | 0.038 | 2.9E-06 | 0.020 | 0.045 | 0.66 |
|  |  |  | rs144669103 |  | T | 0.046 | -0.103 | 0.023 | 5.0E-06 | -0.028 | 0.026 | 0.29 |
|  |  |  | rs145265872 |  | T | 0.046 | -0.107 | 0.023 | 3.0E-06 | 0.041 | 0.027 | 0.12 |
|  |  |  | rs147256205 |  | A | 0.014 | 0.188 | 0.041 | 4.6E-06 | 0.050 | 0.048 | 0.30 |
|  |  |  | rs149251474 |  | G | 0.022 | 0.158 | 0.034 | 2.8E-06 | -0.044 | 0.041 | 0.28 |
|  |  |  |  |  |  |  | Gene-exposure estimate | | | Gene-outcome estimate | | |
| Exposure | Outcome | Threshold genetic instrument | SNP | Original SNP if proxy used (LD R^2^ with original SNP) | Effect allele | Effect allele frequency | beta | SE | p-value | beta | SE | p-value |
|  |  |  | rs149326099 |  | C | 0.036 | -0.115 | 0.026 | 8.6E-06 | 0.047 | 0.029 | 0.11 |
|  |  |  | rs1547668 |  | G | 0.796 | -0.054 | 0.012 | 3.6E-06 | 0.022 | 0.013 | 0.10 |
|  |  |  | rs1552291 |  | G | 0.629 | 0.047 | 0.010 | 1.9E-06 | -0.029 | 0.011 | 0.01 |
|  |  |  | rs17400178 |  | T | 0.071 | 0.084 | 0.019 | 6.6E-06 | -0.013 | 0.022 | 0.55 |
|  |  |  | rs1994140 |  | G | 0.565 | -0.046 | 0.009 | 1.0E-06 | 0.016 | 0.011 | 0.14 |
|  |  |  | rs2087000 |  | C | 0.223 | 0.051 | 0.011 | 8.0E-06 | 0.001 | 0.013 | 0.96 |
|  |  |  | rs208827 |  | G | 0.636 | 0.051 | 0.010 | 2.2E-07 | -0.024 | 0.011 | 0.03 |
|  |  |  | rs2206301 |  | C | 0.625 | -0.050 | 0.010 | 3.2E-07 | 0.001 | 0.011 | 0.96 |
|  |  |  | rs2673609 |  | G | 0.360 | 0.045 | 0.010 | 5.0E-06 | -0.005 | 0.011 | 0.67 |
|  |  |  | rs4772694 |  | T | 0.280 | -0.046 | 0.010 | 8.6E-06 | 0.017 | 0.012 | 0.16 |
|  |  |  | rs618932 |  | G | 0.994 | 0.264 | 0.060 | 9.2E-06 | 0.033 | 0.064 | 0.60 |
|  |  |  | rs62515690 |  | A | 0.015 | 0.199 | 0.043 | 4.6E-06 | 0.021 | 0.045 | 0.64 |
|  |  |  | rs62525086 |  | C | 0.056 | 0.093 | 0.020 | 6.0E-06 | -0.053 | 0.023 | 0.02 |
|  |  |  | rs6437732 |  | T | 0.511 | -0.042 | 0.009 | 6.6E-06 | 0.002 | 0.011 | 0.82 |
|  |  |  | rs72664496 |  | A | 0.066 | -0.085 | 0.019 | 9.3E-06 | -0.004 | 0.022 | 0.85 |
|  |  |  | rs72802225 |  | G | 0.014 | 0.204 | 0.043 | 1.9E-06 | 0.131 | 0.053 | 0.01 |
|  |  |  | rs72835405 |  | T | 0.233 | -0.051 | 0.011 | 6.2E-06 | 0.009 | 0.013 | 0.49 |
|  |  |  | rs7332332 |  | G | 0.497 | -0.044 | 0.009 | 3.5E-06 | -0.002 | 0.011 | 0.83 |
|  |  |  | rs738475 |  | G | 0.259 | 0.051 | 0.011 | 2.5E-06 | 0.000 | 0.013 | 1.00 |
|  |  |  | rs7423933 |  | A | 0.735 | -0.047 | 0.011 | 9.1E-06 | 0.006 | 0.012 | 0.65 |
|  |  |  | rs79278977 |  | T | 0.014 | -0.194 | 0.042 | 3.5E-06 | -0.025 | 0.049 | 0.61 |
|  |  |  | rs865199 |  | G | 0.932 | 0.086 | 0.019 | 4.1E-06 | -0.004 | 0.021 | 0.85 |

*Replication with the 2^nd^ release of UK biobank as the outcome-sample (*n*=67,193 for cigarettes per day / *n*=107,874 for smoking cessation) instead of the TAG consortium (*n*=38,181 / *n*=67,193, respectively).

**Supplementary Table 9.** Cochran's Heterogeneity statistic for Inverse Variance Weighted (IVW) two-sample Mendelian randomization analyses estimating causal effects of smoking initiation on sleep behaviours (sleep duration, chronotype and insomnia).

| Exposure | Outcome | Threshold | N SNPs | Cochran’s heterogeneity  statistic | |
| --- | --- | --- | --- | --- | --- |
|  |  |  |  | Q | *p* |
| Smoking initiation | Sleep duration | *p*<1×10^-5^ | 19 | 15.99 | 0.59 |
| Smoking initiation | Undersleeping | *p*<1×10^-5^ | 19 | 11.74 | 0.86 |
| Smoking initiation | Oversleeping | *p*<1×10^-5^ | 19 | 10.95 | 0.90 |
| Smoking initiation | Chronotype | *p*<1×10^-5^ | 19 | 21.10 | 0.28 |
| Smoking initiation | Insomnia | *p*<1×10^-5^ | 20 | 20.85 | 0.35 |

Cochran’s (Q) statistic provides an indication of heterogeneity between the estimates of the individual SNPs for IVW analyses.

**Supplementary Table 10.** I-squared statistic

| Exposure | Outcome | Threshold | N SNPs |  |
| --- | --- | --- | --- | --- |
|  |  |  |  | I^2^ |
| Smoking initiation | Sleep duration | *p*<1×10^-5^ | 19 | 0.48 |
| Smoking initiation | Undersleeping | *p*<1×10^-5^ | 19 | 0.49 |
| Smoking initiation | Oversleeping | *p*<1×10^-5^ | 19 | 0.49 |
| Smoking initiation | Chronotype | *p*<1×10^-5^ | 19 | 0.48 |
| Smoking initiation | Insomnia | *p*<1×10^-5^ | 20 | 0.48 |

I^2^ quantifies heterogeneity between the genetic variants in an instrument and indicates whether the ’NO Measurement Error’ (NOME) assumption is likely to have been violated. If I^2^ is smaller than 0.9, the NOME assumption may be violated.

**Supplementary Table 11.** MR-Egger intercept, indicating pleiotropy, for two-sample Mendelian randomization analyses estimating causal effects of smoking initiation on sleep behaviours (sleep duration, chronotype and insomnia).

| Exposure | Outcome | Threshold | N SNPs | MR-Egger intercept | |
| --- | --- | --- | --- | --- | --- |
|  |  |  |  | Intercept (95% CI) | *p* |
| Smoking initiation | Sleep duration | *p*<1×10^-5^ | 19 | -0.002 (-0.010 to 0.006) | 0.71 |
| Smoking initiation | Undersleeping | *p*<1×10^-5^ | 19 | 1.00 (0.998 to 1.004) | 0.56 |
| Smoking initiation | Oversleeping | *p*<1×10^-5^ | 19 | 1.00 (0.997 to 1.002) | 0.67 |
| Smoking initiation | Chronotype | *p*<1×10^-5^ | 19 | 0.007 (-0.004 to 0.018) | 0.23 |
| Smoking initiation | Insomnia | *p*<1×10^-5^ | 20 | 0.99 (0.97 to 1.004) | 0.16 |

**Supplementary Table 12.** Cochran's Heterogeneity statistic for Inverse Variance Weighted (IVW) two-sample Mendelian randomization analyses estimating causal effects of sleep behaviours (sleep duration, chronotype and insomnia) on smoking behaviours (initiation, cigarettes per day, smoking cessation).

| Exposure | Outcome | Threshold | N SNPs | Cochran’s heterogeneity statistic | |
| --- | --- | --- | --- | --- | --- |
|  |  |  |  | Q | *p* |
| Sleep duration | Smoking initiation | *p*<5×10^-8^ | 3 | 2.96 | 0.23 |
|  |  | *p*<1×10^-5^ | 23 | 31.03 | 0.10 |
| Sleep duration | Cigarettes per day | *p*<5×10^-8^ | 3 | 1.38 | 0.50 |
|  |  | *p*<1×10^-5^ | 23 | 20.71 | 0.54 |
| Sleep duration | Smoking cessation | *p*<5×10^-8^ | 3 | 1.13 | 0.57 |
|  |  | *p*<1×10^-5^ | 23 | 20.69 | 0.54 |
|  |  |  |  |  |  |
| Undersleeping | Smoking initiation | *p*<1×10^-5^ | 15 | 16.61 | 0.28 |
| Undersleeping | Cigarettes per day | *p*<1×10^-5^ | 15 | 7.64 | 0.91 |
| Undersleeping | Smoking cessation | *p*<1×10^-5^ | 15 | 10.55 | 0.72 |
|  |  |  |  |  |  |
| Oversleeping | Smoking initiation | *p*<1×10^-5^ | 12 | 8.46 | 0.67 |
| Oversleeping | Cigarettes per day | *p*<1×10^-5^ | 12 | 4.44 | 0.96 |
| Oversleeping | Smoking cessation | *p*<1×10^-5^ | 12 | 24.81 | 0.01 |
|  |  |  |  |  |  |
| Chronotype | Smoking initiation | *p*<5×10^-8^ | 8 | 8.26 | 0.31 |
|  |  | *p*<1×10^-5^ | 55 | 51.95 | 0.55 |
| Chronotype | Cigarettes per day | *p*<5×10^-8^ | 8 | 16.14 | 0.02 |
|  |  | *p*<1×10^-5^ | 55 | 76.84 | 0.02 |
| Chronotype | Smoking cessation | *p*<5×10^-8^ | 8 | 2.69 | 0.91 |
|  |  | *p*<1×10^-5^ | 55 | 57.41 | 0.35 |
|  |  |  |  |  |  |
| Insomnia | Smoking initiation | *p*<5×10^-8^ | 1 | - | - |
|  |  | *p*<1×10^-5^ | 16 | 20.24 | 0.16 |
| Insomnia | Cigarettes per day | *p*<5×10^-8^ | 1 | - | - |
|  |  | *p*<1×10^-5^ | 16 | 10.94 | 0.76 |
|  |  | *p*<1×10^-5^* | 38 | 38.15 | 0.42 |
| Insomnia | Smoking cessation | *p*<5×10^-8^ | 1 | - | - |
|  |  | *p*<1×10^-5^ | 16 | 14.00 | 0.53 |
|  |  | *p*<1×10^-5^* | 38 | 41.58 | 0.28 |

Cochran’s (Q) statistic provides an indication of heterogeneity between the estimates of the individual SNPs for IVW analyses. *Replication with the 2^nd^ release of UK biobank as the outcome-sample (*n*=67,193 for cigarettes per day / *n*=107,874 for smoking cessation) instead of the TAG consortium (*n*=38,181 / *n*=67,193, respectively).

**Supplementary Table 13.** I-squared statistic

| Exposure | Outcome | Threshold | N SNPs |  |
| --- | --- | --- | --- | --- |
|  |  |  |  | I^2^ |
| Sleep duration | Smoking initiation | *p*<5×10^-8^ | 3 | - |
|  |  | *p*<1×10^-5^ | 23 | 0.68 |
| Sleep duration | Cigarettes per day | *p*<5×10^-8^ | 3 | - |
|  |  | *p*<1×10^-5^ | 23 | 0.63 |
| Sleep duration | Smoking cessation | *p*<5×10^-8^ | 3 | - |
|  |  | *p*<1×10^-5^ | 23 | 0.65 |
|  |  |  |  |  |
| Undersleeping | Smoking initiation | *p*<1×10^-5^ | 15 | 0.23 |
| Undersleeping | Cigarettes per day | *p*<1×10^-5^ | 15 | 0.10 |
| Undersleeping | Smoking cessation | *p*<1×10^-5^ | 15 | 0.05 |
|  |  |  |  |  |
| Oversleeping | Smoking initiation | *p*<1×10^-5^ | 12 | 0.56 |
| Oversleeping | Cigarettes per day | *p*<1×10^-5^ | 12 | 0.56 |
| Oversleeping | Smoking cessation | *p*<1×10^-5^ | 12 | 0.56 |
|  |  |  |  |  |
| Chronotype | Smoking initiation | *p*<5×10^-8^ | 8 | - |
|  |  | *p*<1×10^-5^ | 55 | 0.36 |
| Chronotype | Cigarettes per day | *p*<5×10^-8^ | 8 | - |
|  |  | *p*<1×10^-5^ | 55 | 0.36 |
| Chronotype | Smoking cessation | *p*<5×10^-8^ | 8 | - |
|  |  | *p*<1×10^-5^ | 55 | 0.36 |
|  |  |  |  |  |
| Insomnia | Smoking initiation | *p*<5×10^-8^ | 1 | - |
|  |  | *p*<1×10^-5^ | 16 | 0.32 |
| Insomnia | Cigarettes per day | *p*<5×10^-8^ | 1 | - |
|  |  | *p*<1×10^-5^ | 16 | 0.26 |
|  |  | *p*<1×10^-5^* | 38 | 0.83 |
| Insomnia | Smoking cessation | *p*<5×10^-8^ | 1 | - |
|  |  | *p*<1×10^-5^ | 16 | 0.28 |
|  |  | *p*<1×10^-5^* | 38 | 0.83 |

I^2^ quantifies heterogeneity between the genetic variants in an instrument and indicates whether the ’NO Measurement Error’ (NOME) assumption is likely to have been violated. If I^2^ is smaller than 0.9, the NOME assumption may be violated. *Replication with the 2^nd^ release of UK biobank as the outcome-sample (*n*=67,193 for cigarettes per day / *n*=107,874 for smoking cessation) instead of the TAG consortium (*n*=38,181 / *n*=67,193, respectively).

**Supplementary Table 14.** MR-Egger intercept, indicating pleiotropy, for two-sample Mendelian randomization analyses estimating causal effects of sleep behaviours (sleep duration, chronotype and insomnia) on smoking behaviours (initiation, cigarettes per day, smoking cessation).

| Exposure | Outcome | Threshold | N SNPs | MR-Egger intercept | |
| --- | --- | --- | --- | --- | --- |
|  |  |  |  | Intercept (95% CI) | *p* |
| Sleep duration | Smoking initiation | *p*<5×10^-8^ | 3 | - | - |
|  |  | *p*<1×10^-5^ | 23 | 1.03 (0.99 to 1.08) | 0.16 |
| Sleep duration | Cigarettes per day | *p*<5×10^-8^ | 3 | - | - |
|  |  | *p*<1×10^-5^ | 23 | -0.04 (-0.28 to 0.20) | 0.76 |
| Sleep duration | Smoking cessation | *p*<5×10^-8^ | 3 | - | - |
|  |  | *p*<1×10^-5^ | 23 | 1.00 (0.95 to 1.05) | 0.92 |
|  |  |  |  |  |  |
| Undersleeping | Smoking initiation | *p*<1×10^-5^ | 15 | 1.02 (0.96 to 1.09) | 0.50 |
| Undersleeping | Cigarettes per day | *p*<1×10^-5^ | 15 | -0.11 (-0.40 to 0.18) | 0.48 |
| Undersleeping | Smoking cessation | *p*<1×10^-5^ | 15 | 1.01 (0.94 to 0.08) | 0.84 |
|  |  |  |  |  |  |
| Oversleeping | Smoking initiation | *p*<1×10^-5^ | 12 | 0.98 (0.96 to 1.00) | 0.11 |
| Oversleeping | Cigarettes per day | *p*<1×10^-5^ | 12 | -0.01 (-0.14 to 0.12) | 0.88 |
| Oversleeping | Smoking cessation | *p*<1×10^-5^ | 12 | 1.04 (0.97 to 1.11) | 0.25 |
|  |  |  |  |  |  |
| Chronotype | Smoking initiation | *p*<5×10^-8^ | 8 | - | - |
|  |  | *p*<1×10^-5^ | 55 | 0.97 (0.96 to 0.98) | 1×10^-5^ |
| Chronotype | Cigarettes per day | *p*<5×10^-8^ | 8 | - | - |
|  |  | *p*<1×10^-5^ | 55 | -0.01 (-0.13 to 0.11) | 0.85 |
| Chronotype | Smoking cessation | *p*<5×10^-8^ | 8 | - | - |
|  |  | *p*<1×10^-5^ | 55 | 0.99 (0.97 to 1.01) | 0.41 |
|  |  |  |  |  |  |
| Insomnia | Smoking initiation | *p*<5×10^-8^ | 1 | - | - |
|  |  | *p*<1×10^-5^ | 16 | 1.01 (0.94 to 1.04) | 0.80 |
| Insomnia | Cigarettes per day | *p*<5×10^-8^ | 1 | - | - |
|  |  | *p*<1×10^-5^ | 16 | 0.17 (-0.08 to 0.41) | 0.20 |
|  |  | *p*<1×10^-5^* | 38 | 0.01 (-0.09 to 0.10) | 0.89 |
| Insomnia | Smoking cessation | *p*<5×10^-8^ | 1 |  | - |
|  |  | *p*<1×10^-5^ | 16 | 1.05 (0.999 to 1.11) | 0.09 |
|  |  | *p*<1×10^-5^* | 38 | 0.98 (0.95 to 1.001) | 0.06 |

*Replication with the 2^nd^ release of UK biobank as the outcome-sample (*n*=67,193 for cigarettes per day / *n*=107,874 for smoking cessation) instead of the TAG consortium (*n*=38,181 / *n*=67,193, respectively).
